# Effects of AI-mediated real-time feedback on motivation and motor skill learning in traditional ethnic sports education: testing self-determination theory mechanisms

**DOI:** 10.3389/fpsyg.2026.1958348

**Published:** 2026-10-16

**Authors:** Yuequan Qin, Yu Yang, Guizhen Chen, Huasheng Huang, Dongyi Wei

**Affiliations:** 1 Physical Education Department, Guangxi University of Chinese Medicine, Nanning, Guangxi, China; 2 First Affiliated Hospital, Guangxi University of Chinese Medicine, Nanning, Guangxi, China

**Keywords:** artificial intelligence, competence, motor skill learning, real-time feedback, self-determination theory, traditional ethnic sports, Zhuang Daoyin

## Abstract

**Introduction:**

Traditional ethnic sports such as Zhuang Daoyin demand precise movement, yet conventional demonstration–imitation–verbal correction limits feedback timeliness and individualization. Artificial intelligence (AI) real-time posture feedback offers a scalable option, but most evaluations emphasize technical outcomes and overlook learning processes. Grounded in self-determination theory (SDT), this study tested whether and how AI real-time feedback improves motivation and motor skill by engaging basic psychological needs, with competence as the hypothesized pathway.

**Methods:**

In a 12-week field experiment, 12 intact classes (307 students; control *n* = 152, AI *n* = 155) were randomly allocated at the class level to an AI real-time feedback group or a traditional teaching group; three instructors each taught two classes per condition. Both groups received identical instruction; conditions differed only in feedback source during practice (RTMPose-based concurrent vs. teacher-led terminal feedback). Need satisfaction, motivational regulation (relative autonomy index, RAI), motor skill, and course satisfaction were assessed at pre- and post-test, and analyzed with linear mixed models and structural equation modeling (lavaan, MLR).

**Results:**

The AI group showed significantly greater gains in competence (*d* = 0.36, *p* = 0.003), autonomy (*d* = 0.40, *p* = 0.001), motor skill (*d* = 0.30, *p* = 0.010), and learning interest (*d* = 0.38, *p* = 0.003), whereas relatedness was unaffected (*p* = 0.974); intraclass correlations were negligible. In the structural model (CFI = 0.94, RMSEA = 0.04, SRMR = 0.04), group predicted competence (*β* = 0.17), competence predicted RAI (*β* = 0.34), and RAI predicted motor skill (*β* = 0.30); the autonomy and relatedness paths were nonsignificant. The effect of AI feedback on motor skill was fully mediated by the competence-to-RAI sequence (indirect *p* = 0.023; direct *p* = 0.097).

**Discussion:**

AI real-time feedback improved motor skill by strengthening competence and supporting motivational internalization, while leaving relatedness unaffected—providing discriminant evidence that the tool acts through its affordances rather than diffuse enthusiasm. Systems enhancing performance and learning should prioritize feedback that makes progress legible, within a human–AI division of labor where teachers provide relational support. In conclusion, AI real-time posture feedback can enhance motivation and motor skill learning by satisfying learners’ competence need.

## 1 Introduction

Traditional ethnic sports are both forms of physical activity and embodied carriers of cultural knowledge, yet their place in university physical education (PE) remains vulnerable. In China, initiatives associated with Healthy China 2030, the preservation of ethnic culture, and Education Informatization 2.0 have created a strong policy rationale for modernizing the teaching of traditional sports while maintaining their cultural integrity (Ministry of Education of the People’s Republic of China, 2018; The State Council of the People’s Republic of China, 2016; Mo et al., 2026). Zhuang Daoyin, a traditional practice that coordinates posture, breathing, rhythm, and expressive movement, illustrates the pedagogical challenge. Its complex movement patterns require learners to recognize subtle discrepancies between demonstrated and performed actions. Conventional instruction follows a demonstration–imitation–verbal correction sequence in which one teacher observes many students and provides feedback after errors have occurred. This model limits the timeliness, frequency, and individual specificity of correction, contributing to uneven learning experiences and making it difficult to sustain students’ motivation (Mo et al., 2026). In a context where qualified instructors are scarce and the transmission of traditional movement skills depends heavily on face-to-face apprenticeship, scalable pedagogical tools are urgently needed.

Artificial intelligence (AI) offers a possible response. Computer vision and wearable sensing applications in PE can be grouped into three functional categories: (a) automated performance scoring systems that evaluate movement quality and assign grades (Zhu et al., 2020; Jiang et al., 2024), (b) personalized training-plan generators that adapt exercise prescriptions to individual profiles (Li et al., 2025; Wang and Wang, 2024), and (c) real-time formative feedback systems that provide moment-to-moment postural guidance during practice (Hsia et al., 2024; Lin et al., 2022). Each category engages the learner differently. Scoring and ranking operate primarily through external evaluation, which may evoke social comparison. Personalized plans adapt the content but the feedback loop remains slow—they change between sessions, not within them. Real-time feedback, by contrast, closes the loop inside a single practice bout: the learner executes a movement, the system detects deviations from the target form, and corrective cues are displayed within seconds (Huang et al., 2023). It is this immediacy that makes real-time feedback well suited for SDT-based investigation, because it allows the learner to experience competence gains and adjust volitionally during the act of learning itself. The present study focuses on this third category, using an RTMPose-based real-time feedback system (Jiang et al., 2023; Wei et al., 2026a) specifically designed for the traditional ethnic sport of Zhuang Daoyin.

The psychological experience of receiving algorithmic feedback has received particularly limited attention. A learner may interpret real-time posture feedback as evidence of growing mastery, as an opportunity to take control of practice, or as an external form of surveillance—and these interpretations carry different motivational consequences. Our prior work addressed a related but distinct question. Using the same RTMPose-based system, Zhuang Daoyin course, and institutional context, Wei et al. (2026a) showed that AI feedback improved motor skill through behavioral-cognitive mediators: increased self-regulated learning and practice time. That study established that AI feedback changes what students do; the present study asks whether and how it changes how students feel—specifically, whether AI feedback satisfies the basic psychological needs that self-determination theory identifies as the foundations of autonomous motivation.

This distinction matters for both theory and design. A system that raises technical scores without supporting adaptive motivation may have limited educational value once the novelty wears off. Conversely, mechanism-based evidence can guide the design of AI feedback features that improve both performance and the quality of the learning experience (Du and Qin, 2025; Lazowski and Hulleman, 2016). Yet the prevailing approach in AI-supported PE has been to compare post-intervention outcomes and attribute group differences globally to “the AI system.” What is missing is a theoretically specified account of which psychological functions an AI tool actually serves, which needs it can and cannot satisfy, and how those need-specific effects translate into motivation and learning.

Self-determination theory (SDT) provides the framework for such an account. The theory holds that high-quality motivation depends on satisfying three basic psychological needs: autonomy (volition and self-endorsement), competence (effectiveness and growing mastery), and relatedness (mutual care and belonging) (Ryan and Deci, 2000, 2017; Deci and Ryan 2000). Motivation varies along a continuum of relative autonomy, from controlled forms (external and introjected regulation) to autonomous forms (identified regulation and intrinsic motivation), summarized by the relative autonomy index (RAI) (Grolnick and Ryan, 1989; Vallerand, 1997). In PE settings, environments that satisfy these needs promote autonomous motivation, which in turn predicts engagement, effort, learning, and positive affect (Cheon et al., 2012; Ntoumanis and Standage, 2009; Standage et al., 2003). Critically, instructional interventions influence outcomes only to the extent that they engage students’ basic psychological needs. Wei et al. (2026b) demonstrated this complete-mediation pattern in traditional Chinese university PE: the effect of responsibility-granting pedagogy on course satisfaction was fully transmitted through need satisfaction and autonomous motivation. This principle—that a tool’s educational value depends on its capacity to satisfy specific needs—directly informs the design of the present study.

Extending this logic to AI real-time feedback generates differentiated predictions, not a blanket expectation that technology will satisfy every need equally. The key theoretical move is to map the specific affordances of AI posture feedback onto the three SDT needs. Real-time visual signals about movement deviation and improvement make progress legible and correction attainable—functions that directly support competence. The opportunity to practice at one’s own pace, review individualized improvement reports, and select areas for further work may also support autonomy. Posture detection, however, has no interpersonal dimension: it records skeleton coordinates, not social connection. It should therefore not strengthen relatedness, which depends on teacher–student rapport and peer interaction. These differentiated predictions are the empirical signature of a theoretical mechanism. If AI feedback improves all outcomes through undifferentiated enthusiasm or Hawthorne effects, all three needs would rise together. If instead the tool operates through its specific affordances, competence should be the primary bridge to autonomous motivation, autonomy may play a secondary role, and relatedness should show no effect. A null relatedness pathway is thus not a limitation to be explained away but a discriminant test: a non-significant relatedness result would confirm that the motivational impact of AI feedback is functionally specific rather than a diffuse positivity bias.

## 2 Materials and methods

### 2.1 Study design

This study employed a two-group, pre-test/post-test controlled field experiment over a 12-week semester, with intact teaching classes randomly allocated to conditions at the cluster (class) level. Twelve intact teaching classes from a university public PE course were randomly allocated to either the AI real-time feedback group (AI group; 6 classes) or the traditional teaching control group (control group; 6 classes). Because students were enrolled in intact administrative classes, random allocation was performed at the class (cluster) level: the 12 classes were assigned to the two conditions using a computer-generated random sequence. To prevent instructor-level confounding, three qualified instructors delivered the intervention, with each instructor teaching two AI classes and two control classes (a crossed design), so that instructor identity was balanced across the two conditions. Pre-test assessments were administered during Week 0 prior to the intervention. The 12-week teaching intervention ran from Week 1 through Week 12, during which both groups received identical course content (Zhuang Daoyin “He Xu Le” five-part, 10-movement sequence) with the same 90-min weekly sessions. Post-test assessments were conducted at the end of Week 12. The sole difference between the two conditions was the source of corrective feedback during the practice phase: the AI group received real-time posture-recognition feedback via the RTMPose-based system (Wei et al., 2026a) (concurrent feedback), whereas the control group received teacher-led verbal correction (terminal feedback). The study flow is illustrated in Figure 1. This cluster randomized controlled trial is reported in accordance with the CONSORT 2010 statement and its extension for cluster randomized trials (Campbell et al., 2012).

**Figure 1:**
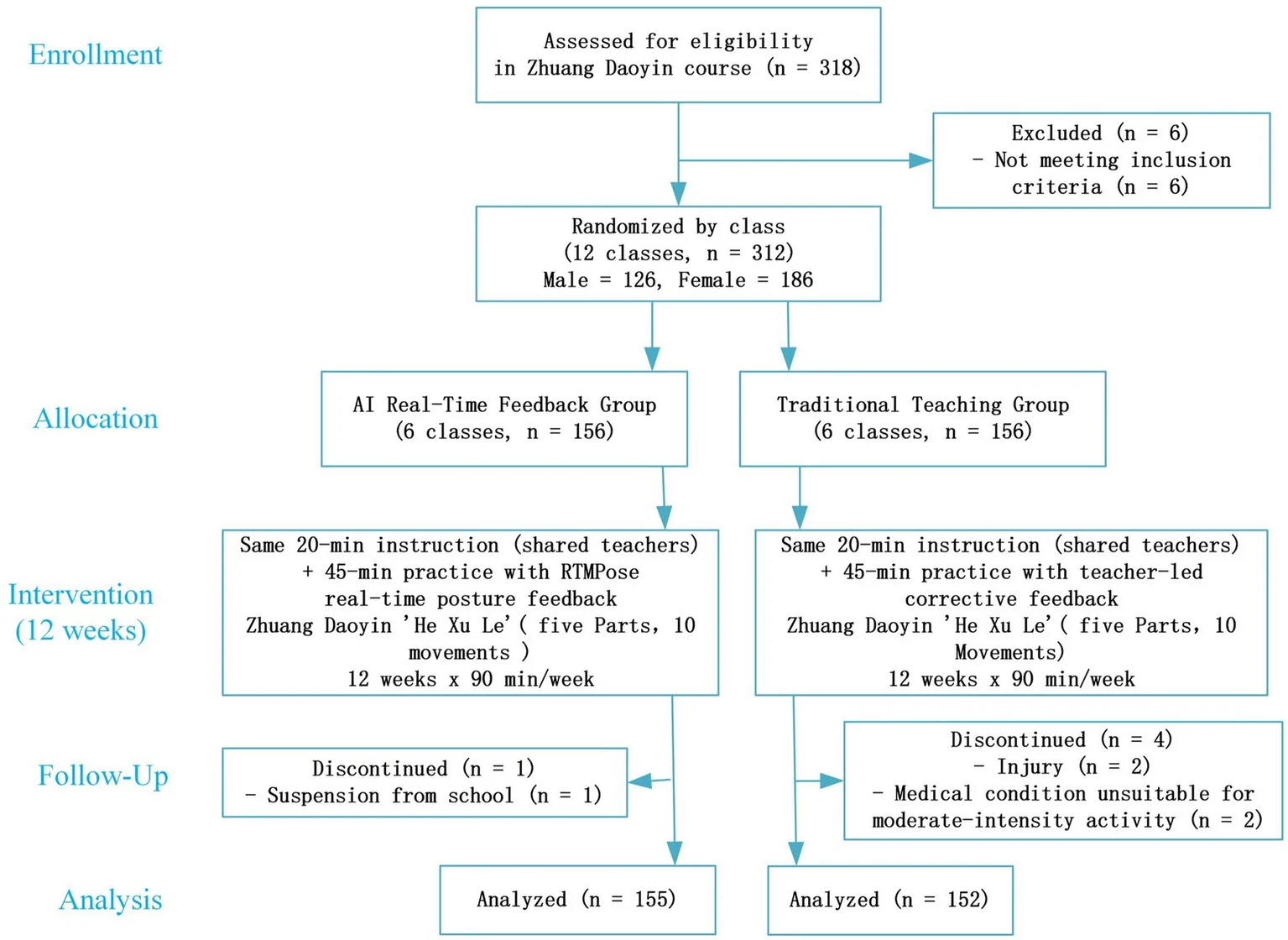
CONSORT flow diagram of participant enrollment, class randomization, allocation, follow-up, and analysis in the randomized controlled trial comparing AI real-time posture feedback versus traditional teacher-led corrective feedback during a 12-week Zhuang Daoyin “He Xu Le” intervention.

### 2.2 Participants

Participants were second-year undergraduate students enrolled in a Zhuang Daoyin public PE elective course at a university of Chinese medicine in Guangxi, China. Inclusion criteria were: (a) full-time second-year enrollment, (b) no prior formal training in Zhuang Daoyin or similar traditional ethnic sports, and (c) voluntary informed consent. Exclusion criteria were: (a) self-reported musculoskeletal injury or medical condition contraindicating moderate physical activity, (b) concurrent enrollment in another structured exercise intervention, and (c) inability to attend at least 80% of scheduled class sessions.

An *a priori* power analysis was conducted using G*Power 3.1 (Faul et al., 2007) for a repeated-measures ANCOVA with two groups, two measurement occasions, a medium effect size (Cohen’s *f* = 0.20), α = 0.05, and power = 0.80. The minimum required sample was 200 participants; because randomization was performed at the class level, this target was inflated by the design effect to account for clustering (Kish, 1965). A total of 318 students across 12 intact classes were assessed for eligibility; 6 did not meet the inclusion criteria and were excluded, yielding 312 participants across the randomized classes (126 male, 186 female). During the intervention, 5 participants discontinued (control group: 4—2 injuries and 2 medical conditions contraindicating moderate-intensity activity; AI group: 1—suspension from school). The final analytic sample comprised 307 participants (control group: *n* = 152; AI group: *n* = 155) who completed both pre-test and post-test assessments.

### 2.3 Ethics approval

The study was approved by the Ethics Committee of Guangxi University of Chinese Medicine (approval number: GXTCMU-EC KS20251210-27, dated February 10, 2025). The study was conducted in accordance with the ethical principles for medical research involving human subjects set forth in the Declaration of Helsinki (World Medical Association, 2013). Prior to enrollment, all participants were fully informed of the study’s purpose, procedures, expected duration, potential risks and benefits, and their right to withdraw at any time without any academic or other penalty. Written informed consent was obtained from every participant before data collection. Participation was entirely voluntary and had no bearing on students’ course grades. All data were anonymized and stored securely with access restricted to the research team, thereby safeguarding participant confidentiality and privacy. The intervention involved only moderate-intensity traditional health-care exercises and posed minimal foreseeable risk to participants.

### 2.4 Intervention

#### 2.4.1 Overview and common session structure

Both groups completed a 12-week intervention (one 90-min session per week) covering the identical Zhuang Daoyin “He Xu Le” five-part, 10-movement sequence. Each weekly session followed the same five-phase structure for both groups: warm-up (10 min), instruction and review of new movements (20 min), practice with corrective feedback (45 min), integrated routine practice (10 min), and cool-down with summary (5 min). The two conditions were identical in every phase except the source of corrective feedback during the 45-min practice phase: the AI group practiced with real-time posture-recognition feedback (concurrent feedback), whereas the control group practiced with teacher-led corrective feedback (terminal feedback). Warm-up, new-movement instruction, integrated practice, and cool-down were delivered identically to both groups, ensuring equivalent time-on-task and content dosage. The 12-week intervention comprised a total of 12 weekly sessions (one 90-min session per week). Mean attendance was high and comparable across conditions (AI group: 94.2%; control group: 94.6%).

#### 2.4.2 New-movement instruction (identical for both groups)

During the 20-min instruction phase, the same standardized lesson plan and demonstration protocol were used for both groups. The same crossed-instructor arrangement described in Section 2.1 was applied. The domain expert (first author) trained all instructors and defined the criterion movement templates to ensure consistency of demonstration and terminology. All instruction sessions were video-recorded and periodically reviewed for fidelity.

#### 2.4.3 AI real-time feedback group (practice phase)

The AI feedback system was built on the RTMPose (Real-Time Multi-Person Pose Estimation) posture-recognition architecture, which employs a CSPNeXt backbone with SimCC coordinate classification to detect 133 body keypoints in real time (Jiang et al., 2023; Wei et al., 2026a). A standard webcam (1920 × 1,080 resolution, 30 fps) was positioned on a tripod 2.5 m directly in front of the practice area, with the camera lens aligned to the practitioner’s chest height so that the full body remained centered in the frame. The system processed each video frame with a mean latency of approximately 120 ms, comparing the extracted skeleton sequence against a reference database constructed from 28 expert-validated action templates. Feedback was displayed on a 24-inch monitor placed on the same side as the practitioner, 0.7–1.0 m away and angled 20°–30° off the frontal axis and took two complementary forms: (a) a skeleton overlay with the student’s current posture superimposed on the expert reference, with deviation vectors drawn at key joints, and (b) a color-coded heatmap on an adjacent body diagram indicating regions with the largest alignment errors (red: large deviation; yellow: moderate; green: within acceptable range). During the 45-min practice phase, students practiced individually while connected to the system and received concurrent visual feedback, adjusting their movements and repeating each attempt as the system updated on every frame. Throughout this phase, the three instructors circulated only to ensure safety and did not provide technical corrections, so that the AI system served as the sole source of corrective feedback for this group. At the end of each session, an individualized learning report summarizing movement-specific accuracy scores and key areas for improvement was generated automatically. A complete technical description of the system architecture, training data, and validation is provided in Wei et al. (2026a) (Figure 2).

**Figure 2:**
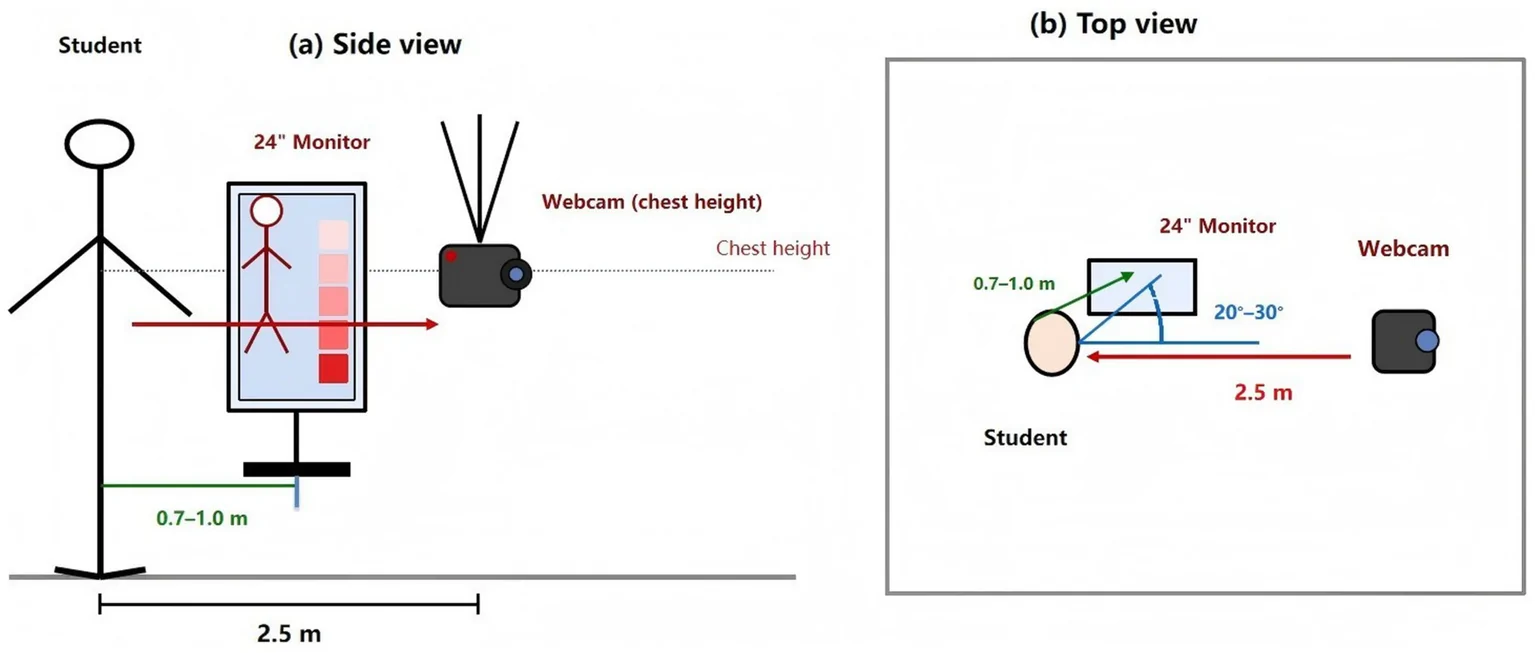
AI practice workstation layout: **(a)** side view and **(b)** top view. The webcam is mounted on a tripod 2.5 m directly in front of the practitioner, aligned to chest height; the 24-inch monitor is placed on the same side, 0.7–1.0 m away and angled 20°–30° off the frontal axis, showing skeleton overlay and heatmap feedback.

#### 2.4.4 Control group (practice phase)

During the same 45-min practice phase, students in the control group practiced the identical movements while the three instructors circulated and delivered teacher-led verbal corrective feedback following a standardized correction protocol (terminal feedback). No AI-based posture feedback or digital movement analysis was provided to this group.

#### 2.4.5 Course content equivalence

Both groups studied the identical Zhuang Daoyin “He Xu Le” sequence, which consists of five parts corresponding to the “Three Channels and Two Routes” (Grain Channel, Water Channel, Qi Channel, Dragon Route, and Fire Route) and comprises 10 movements in total (Table 1). Total contact hours, movement content, learning objectives, and session structure were identical across the two groups; the only difference was the source of corrective feedback during the practice phase.

**Table 1:** The 10 movements of Zhuang Daoyin “He Xu Le”, organized by the five parts (三道两路, Three Channels and Two Routes).

Part (三道两路 Three Channels and Two Routes)	Movement No.	Movement (Chinese)	Movement (Pinyin)
Grain Channel 谷道 · 岩居谷饮 Yanju Guyin (dwelling by rocks, drinking from valleys)	1–1 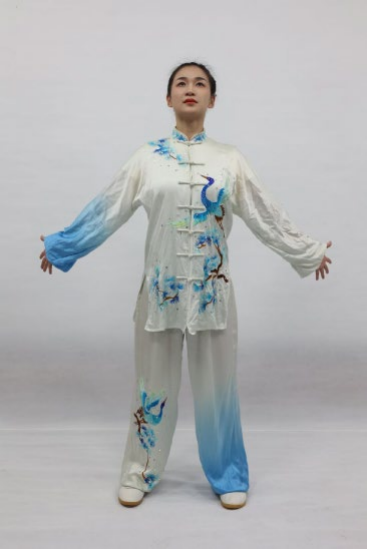	纳谷式	Nagu Shi
	1–2 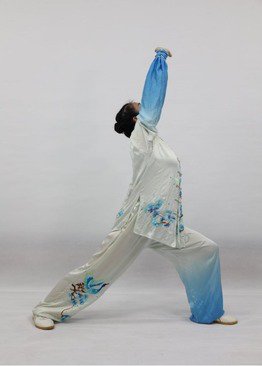	运谷式	Yungu Shi
Water Channel 水道 · 水落归漕 Shuiluo Guicao (water returning to its course)	2–1 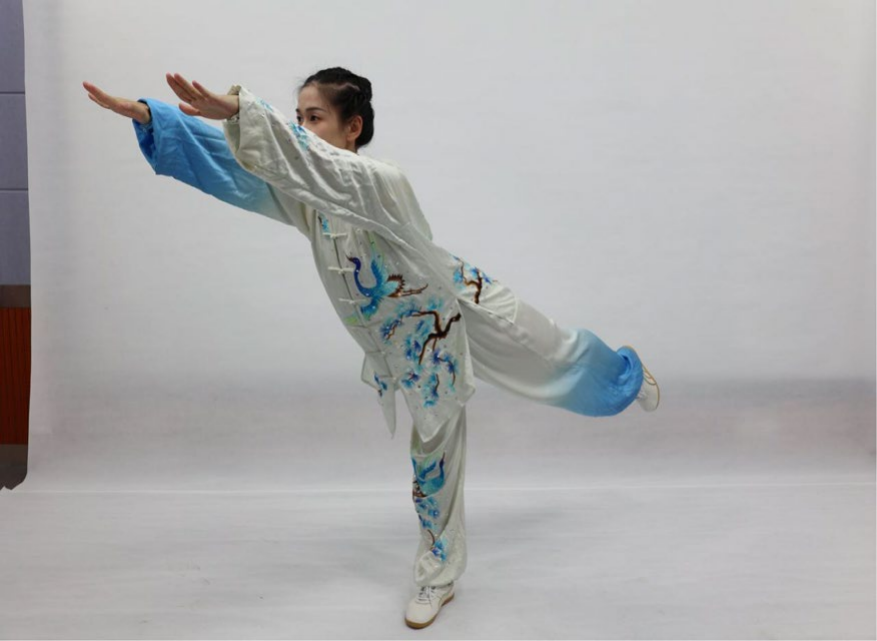	蓄水式	Xushui Shi
	2–2 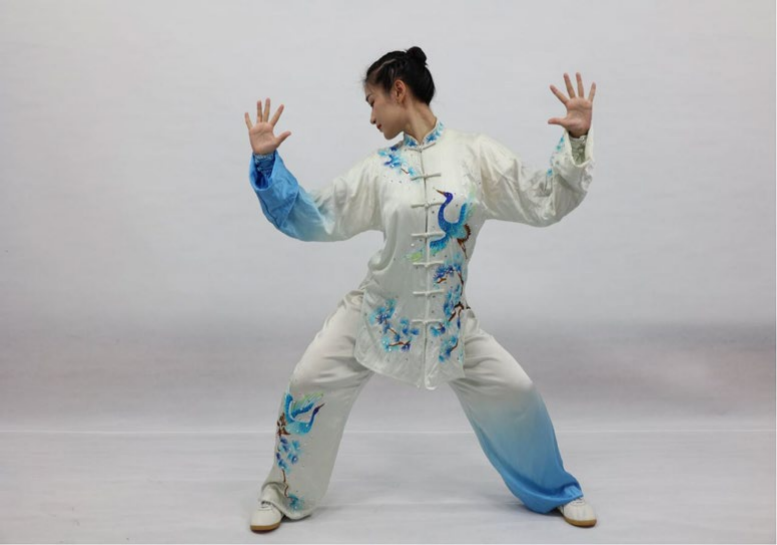	戏水式	Xishui Shi
Qi Channel 气道 · 移气养体 Yiqi Yangti (moving qi to nurture the body)	3–1 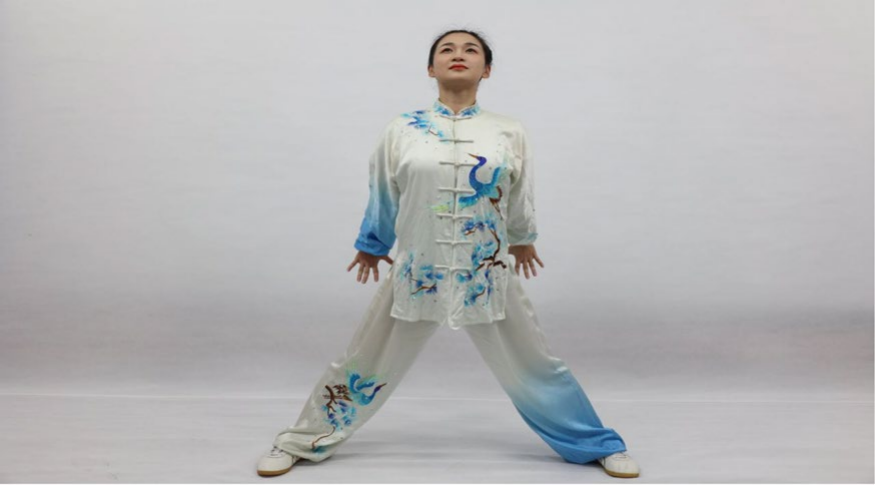	导气式	Daoqi Shi
	3–2 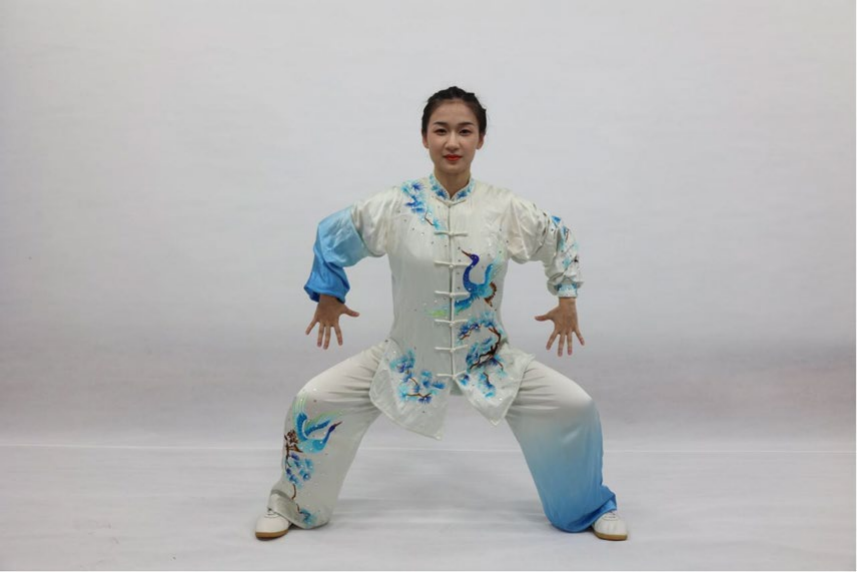	调气式	Tiaoqi Shi
Dragon Route 龙路 · 龙御百川 Longyu Baichuan (the dragon commanding all rivers)	4–1 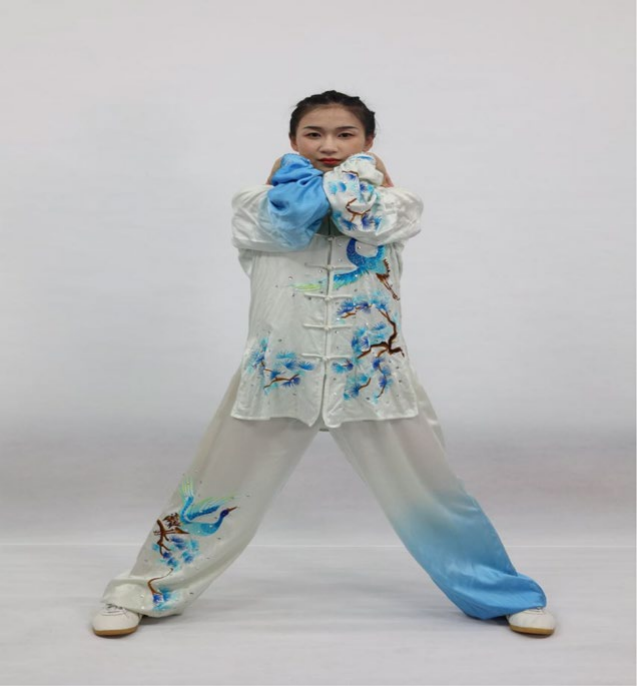	御龙式	Yulong Shi
	4–2 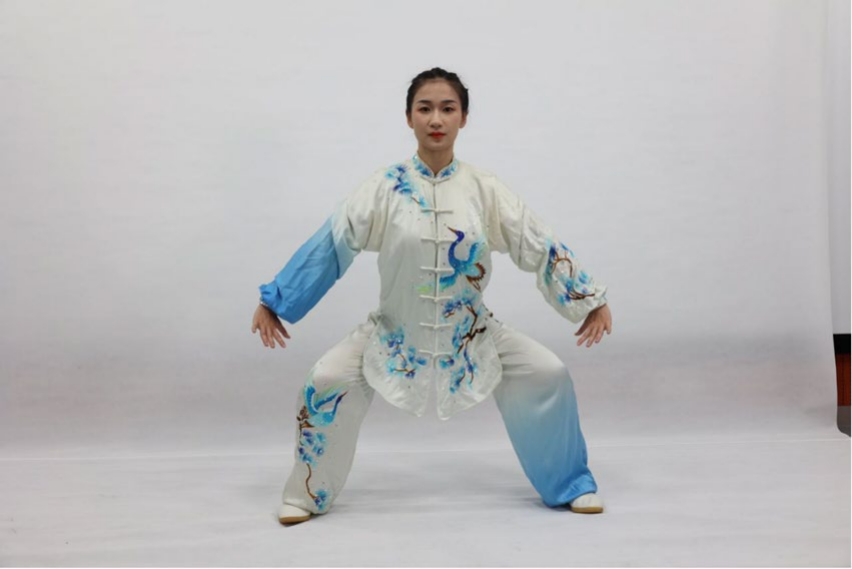	游龙式	Youlong Shi
Fire Route 火路 · 薪火相传 Xinhuo Xiangchuan (passing the flame onward)	5–1 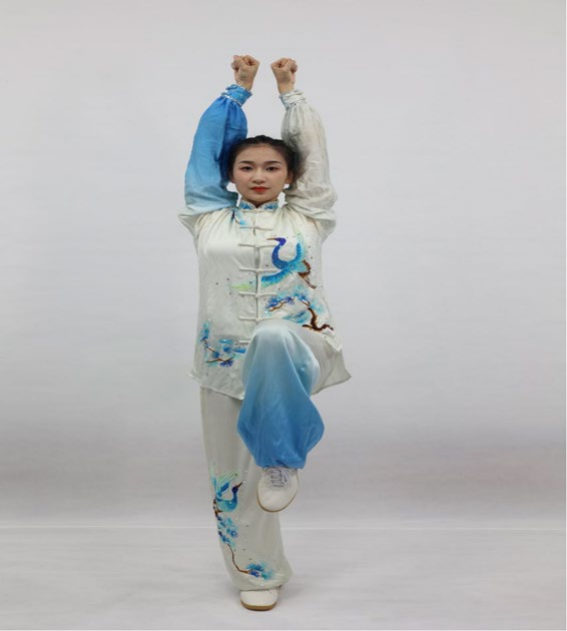	生火式	Shenghuo Shi
	5–2 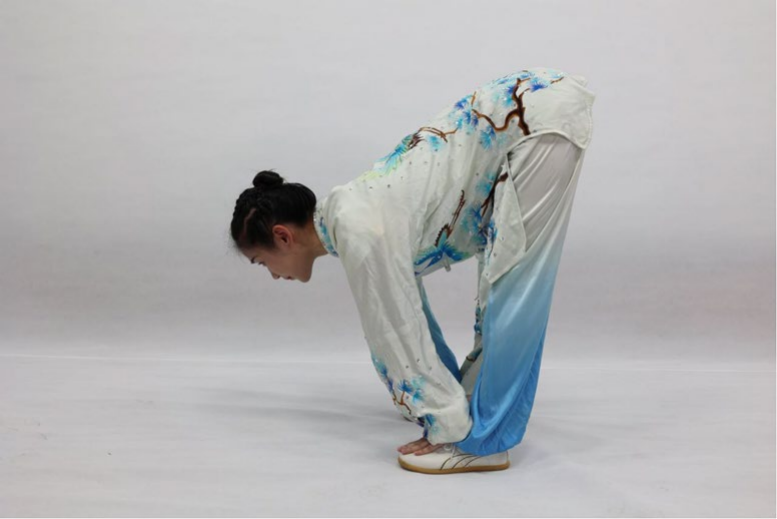	传火式	Chuanhuo Shi

The warm-up (起势) and cool-down (收势) postures are performed before and after the 10 movements and are not included in the numbering.

#### 2.4.6 Treatment fidelity assessment

To minimize contamination across conditions in the crossed-instructor design, three structural safeguards were implemented. First, the domain expert (first author) trained all instructors before the experiment and conducted biweekly fidelity checks by reviewing randomly sampled classroom recordings to verify that the verbal feedback delivered in the control condition adhered to the standardized correction protocol (i.e., did not incorporate AI-style biomechanical language). Second, the AI feedback tablet was removed from the practice area during control-group sessions, so students in the control condition had no exposure to the AI system at any point during the semester. Third, the identical lesson plan and movement sequence were used for both groups (Section 2.4.5), and instructors followed a scripted feedback lexicon that mirrored the AI feedback content in the experimental group but was delivered verbally, without real-time pose visualization. Although formal treatment fidelity coding with independent blind raters was not conducted, these safeguards together reduce the risk of treatment diffusion and ensure that the AI and control conditions differed systematically along the intended dimension of real-time, individual-level posture feedback.

### 2.5 Measures

#### 2.5.1 Motor skill performance (primary outcome)

Motor skill performance was assessed at both pre-test and post-test using a dual-rating procedure. Two experienced Zhuang Daoyin instructors, blinded to group assignment and assessment timing, independently rated each participant’s performance on two sub-dimensions: movement specification (accuracy of posture and transition) and performance level (fluency, rhythm, and expression); these two sub-dimension scores were summed into a total skill score. These two dimensions were derived from established practice in assessing traditional Chinese health-preservation exercise, which conventionally distinguishes form/correctness from expression/fluency; the dimensions were operationalized by a domain expert and anchored to the RTMPose criterion templates. Ratings were based on video recordings of each participant performing the full 10-movement sequence. Inter-rater reliability was assessed using the intraclass correlation coefficient (ICC) for absolute agreement based on a two-way random-effects model (ICC [2,2]; Koo and Li, 2016). The ICC values at pre-test and post-test are reported in Section 3.1. In addition, the AI system itself generated an automatic total score for each participant at post-test, used as a convergent validity check against the human ratings. The complete scoring rubric for motor skill performance is presented in Table 2. Each sub-dimension was rated on a 5-point scale with anchors defined as follows.

**Table 2:** Scoring rubric for the motor skill performance assessment.

Score	Movement Specification (accuracy of posture and transition)	Performance Level (fluency, rhythm, and expression)
5 – Excellent	Posture and transitions accurately match the expert template at every stage; deviations are imperceptible.	Extremely fluent and rhythmic; fully coordinated and highly expressive performance.
4 – Good	Posture and transitions closely match the template; only minor, self-corrected deviations appear.	Fluent and rhythmic with slight hesitation at transitions; good overall expression.
3 – Fair	Posture and transitions approximate the template but show visible deviations at some stages.	Moderately fluent with occasional interruptions; rhythm partially maintained; some expressiveness.
2 – Poor	Posture and transitions deviate clearly from the template; several stages are inaccurate.	Hesitant and uneven rhythm; frequent interruptions; limited expressiveness.
1 – Very poor	Posture and transitions fail to match the template; the sequence is largely unrecognizable.	Disjointed and arrhythmic; no sustained flow or expression.

Movement specification and performance level were each scored on a 5-point scale; the total skill score was the sum of the two sub-dimension scores (range 2–10). Ratings were assigned independently by two instructors blinded to group and time; discrepancies were resolved by consensus.

#### 2.5.2 Basic psychological need satisfaction

Basic psychological need satisfaction was measured using a 12-item scale adapted from Wang and Yu (2019) and Chen et al. (2015). The scale comprises three 4-item subscales corresponding to the three SDT needs: autonomy (e.g., “I feel that the exercises I perform match my ability level”), competence (e.g., “I can effectively complete the movements”), and relatedness (e.g., “I feel I can communicate openly with my peers”). All items were rated on a 5-point Likert scale ranging from 1 (strongly disagree) to 5 (strongly agree). In the present sample, internal consistency was acceptable for all subscales: autonomy (*α* = 0.794), competence (*α* = 0.797), and relatedness (*α* = 0.831).

#### 2.5.3 Motivational regulation

Motivational regulation was assessed using a 16-item scale adapted from the Perceived Locus of Causality (PLOC) measure for physical education (Goudas et al., 1994; Guay et al., 2000). The scale captures four behavioral regulation types with 4 items each: intrinsic motivation (e.g., “Because I enjoy learning Zhuang Daoyin”), identified regulation (e.g., “Because performing well in this course is important to me”), introjected regulation (e.g., “Because I would feel bad about myself if I did not try hard”), and external regulation (e.g., “Because I want the teacher to think I am a good student”). All items were rated on a 5-point Likert scale from 1 (strongly disagree) to 5 (strongly agree). The relative autonomy index (RAI) was computed using the standard weighting formula: RAI = (3 × intrinsic motivation) + (2 × identified regulation) − (1 × introjected regulation) − (3 × external regulation) (Grolnick and Ryan, 1989; Ryan and Connell, 1989; Vallerand, 1997). Higher RAI scores indicate more self-determined motivation.

#### 2.5.4 Course satisfaction (secondary outcome)

Course satisfaction was measured using a 5-item scale adapted from Shi (2010). Items (e.g., “Overall, I find the Zhuang Daoyin course enjoyable”) were rated on a 5-point Likert scale from 1 (strongly disagree) to 5 (strongly agree). Higher scores indicate greater satisfaction with the course experience. Internal consistency in the present sample was excellent (*α* = 0.907).

#### 2.5.5 A learning interest, engagement, concentration, and vitality

All four scales demonstrated acceptable internal consistency in the present sample and were treated as observed variables in outcome analyses. Correlations among these measures and the primary outcome variables are reported in the Supplementary materials.

**Vitality** was measured using a 3-item scale adapted from the Subjective Vitality Scale (Ryan and Frederick, 1997). Items (e.g., “I felt alive and vital during the course,” “I had energy and spirit”) capture the subjective experience of energy and aliveness. All items were rated on a 5-point Likert scale from 1 (strongly disagree) to 5 (strongly agree). Internal consistency was good (*α* = 0.841). These four measures were administered at both pre-test and post-test as secondary indicators of engagement quality and psychological experience accompanying the intervention.

**Concentration** was assessed using a 3-item scale adapted from the attention subscale of the IMI (McAuley et al., 1989). Items (e.g., “I was completely focused on the movements during practice”) were rated on a 5-point Likert scale from 1 (strongly disagree) to 5 (strongly agree). Higher scores indicate greater concentration during the learning activity. Internal consistency was acceptable (*α* = 0.798).

**Engagement** was measured using a 5-item scale adapted from the engagement subscale of the Utrecht Work Engagement Scale for Students (UWES-S; Schaufeli et al., 2002). Items (e.g., “When I am practicing, I feel bursting with energy,” “I am immersed in my practice”) capture the behavioral intensity and absorption facets of engagement. All items were rated on a 5-point Likert scale from 1 (strongly disagree) to 5 (strongly agree), with higher scores reflecting stronger engagement. Internal consistency was good (*α* = 0.873).

**Learning interest** was assessed using a 4-item scale adapted from the Intrinsic Motivation Inventory (IMI) interest/enjoyment subscale (McAuley et al., 1989; Ryan, 1982). Items (e.g., “I enjoyed the Zhuang Daoyin course very much,” “I would describe this course as very interesting”) were rated on a 5-point Likert scale from 1 (strongly disagree) to 5 (strongly agree). Higher scores indicate greater interest in the learning content. Internal consistency was excellent (*α* = 0.916).

To situate these measures within the SDT framework: basic psychological need satisfaction (autonomy, competence, relatedness) and motivational regulation (summarized by the relative autonomy index, RAI) constitute the core SDT mechanism at the heart of our hypotheses, operationalizing the needs-and-motivation pathway theorized to link AI feedback to outcomes. The remaining self-report measures—learning interest, engagement, concentration, and vitality—are not additional theoretical constructs but proximal, downstream manifestations of high-quality (autonomous) motivation, included to triangulate the motivational pathway; course satisfaction is a broader evaluative outcome reflecting the overall learning experience. Each measure therefore occupies a specific and theoretically justified role in the model.

#### 2.5.6 Covariates

Demographic information including age, sex, and prior exercise experience was collected at pre-test via a brief self-report questionnaire. Pre-test motor skill scores and pre-test psychological scale scores were included as covariates in all outcome analyses to control for baseline individual differences.

### 2.6 Procedures

At Week 0, all participants completed the pre-test assessment battery in a single session: the demographic questionnaire, the basic psychological need satisfaction scale, the motivational regulation scale, and the motor skill performance test (video-recorded for later blind rating). During Weeks 1–12, participants attended their assigned weekly 90-min Zhuang Daoyin class. Attendance was recorded at each session. At the end of Week 12, all participants completed the post-test assessment battery, identical to the pre-test; the course satisfaction scale was administered at both pre-test and post-test. All questionnaires were administered in paper-and-pencil format in a quiet classroom setting under the supervision of a research assistant who was not involved in the intervention delivery. Motor skill video recordings were coded by the two blinded raters within 2 weeks of data collection completion.

### 2.7 Statistical analysis

Data were analyzed using R (version 4.3.3). The significance level was set at *α* = 0.05 (two-tailed) for all analyses, except where bootstrap-derived confidence intervals were used. Preliminary analyses included descriptive statistics, independent samples *t*-tests (or χ^2^ tests for categorical variables) to examine baseline equivalence between groups, and Pearson correlations among all study variables. Because random allocation was performed at the class level, the intraclass correlation coefficient (ICC) was computed for each primary outcome to quantify the proportion of variance attributable to class-level clustering, and the corresponding design effect was reported.

For Hypothesis 1 (group differences in competence, autonomous motivation, and motor skill), the clustered structure of the data (students nested within classes) was explicitly modeled. Linear mixed models (LMM) were fitted with Group, Time, and the Group × Time interaction as fixed effects and the corresponding pre-test score as a covariate, and with random intercepts for class and participant to account for the class-level allocation and the repeated-measures structure. The models were fitted with the lme4 and lmerTest packages using restricted maximum likelihood estimation, with degrees of freedom approximated by the Satterthwaite method; this approach is robust to missing data under the missing-at-random assumption (Verbeke and Molenberghs, 2000). As a complementary summary, one-way ANCOVAs were also conducted for each post-test outcome with group as the between-subjects factor and the corresponding pre-test score as the covariate, with effect sizes reported as partial eta-squared (η^2^p); given the clustered design, these ANCOVA results were interpreted as supplementary to the mixed-model estimates.

For Hypotheses 2–4 (mediation pathways), structural equation modeling (SEM) was performed using the lavaan package with robust maximum likelihood estimation (MLR). The measurement model included latent factors for autonomy, competence, relatedness, and motivational regulation (four subscale scores as indicators loading on RAI). The structural model specified: (a) direct paths from group assignment (dummy-coded: 0 = control, 1 = AI) to the three need satisfaction latent factors; (b) paths from each need satisfaction factor to RAI; (c) a path from RAI to post-test motor skill; (d) a path from RAI to course satisfaction; and (e) direct paths from group to motor skill and course satisfaction. Pre-test motor skill scores and pre-test need satisfaction scores were included as covariates. Model fit was evaluated using the comparative fit index (CFI), Tucker–Lewis index (TLI), root mean square error of approximation (RMSEA), and standardized root mean square residual (SRMR). Acceptable fit was defined as CFI ≥ 0.90, TLI ≥ 0.90, RMSEA ≤ 0.08, and SRMR ≤ 0.08 (Hu and Bentler, 1999).

Indirect effects were tested using bootstrap procedures with 5,000 resamples and bias-corrected 95% confidence intervals (Preacher and Hayes, 2008). A specific indirect effect was considered statistically significant if the 95% CI did not include zero. Total, direct, and indirect effects were decomposed to evaluate the extent of mediation. The competence pathway, autonomy pathway, and relatedness pathway were estimated and compared as separate specific indirect effects. The sequential chain mediation (Group → Competence → RAI → Outcome) was tested as the primary hypothesis-driven indirect path. Because the SEM framework does not directly accommodate the three-level clustering structure (students nested within classes, classes nested within instructors), two approaches were used to address potential non-independence. First, class-level intraclass correlation coefficients (ICCs) for all mediator and outcome variables were verified to be negligible (all ICCs ≤ 0.03; see Section 3.1). Second, as a sensitivity check, the key SEM paths were re-estimated with cluster-robust standard errors using the class identifier as the clustering variable (via the lavaan package with the “cluster” argument), which adjusts standard errors for within-class correlation without altering point estimates. The cluster-robust standard errors were nearly identical to the model-based standard errors for all focal paths (e.g., the competence → RAI path: cluster-robust SE = 0.049 vs. model-based SE = 0.047), confirming that clustering did not meaningfully bias the inferences drawn from the single-level SEM. This approach—low ICCs plus cluster-robust sensitivity checks—is recommended when the number of clusters is insufficient for multilevel SEM (Hox et al., 2017; Muthén, 1994).

Sensitivity analyses included: (a) per-protocol analysis excluding participants with attendance below 80%; (b) testing an alternative model in which all three need factors directly predicted motor skill and satisfaction without passing through RAI; (c) multi-group SEM to examine whether the mediation structure was invariant across sex; (d) re-estimation of the key group-difference tests using cluster-robust standard errors with class as the clustering unit to confirm robustness to the clustered allocation; and (e) re-estimation of the structural model using the conventional weighted-composite RAI as a manifest variable, in parallel with the latent RAI specification, to confirm that the mediation pattern did not depend on the modeling approach. In all sensitivity analyses, the core mediation findings were directionally unchanged. Within the SEM framework, missing data were handled using full information maximum likelihood (FIML; Enders and Bandalos, 2001) under the missing-at-random assumption. For the mixed-model and ANCOVA analyses, participants with missing post-test outcome data (*n* = 5; 1.6%) were excluded, yielding a final analytic sample of *N* = 307. Sensitivity analyses confirmed that FIML-based and complete-case SEM estimates produced equivalent results, consistent with the very low rate of missing data.

## 3 Results

### 3.1 Preliminary analyses and baseline equivalence

Of the 312 randomized participants, 307 (control group, *n* = 152; AI real-time feedback group, *n* = 155) completed both the pre-test and post-test assessments and were included in the analyses. Participants had a mean age of 19.4 years (SD = 1.13). Independent-samples t-tests and chi-square tests indicated that the two groups did not differ significantly at baseline on any demographic or outcome variable (all *p* > 0.05; Table 3), confirming baseline equivalence between conditions. Because random allocation was performed at the class level, the intraclass correlation coefficient (ICC) at the class level was computed for each primary outcome; all class-level ICCs were negligible (≤0.03), indicating minimal between-class clustering and supporting the use of individual-level effect estimates. Inter-rater reliability of the motor skill assessment, based on a two-way random-effects, absolute-agreement ICC (2,2), was excellent at both pre-test (ICC = 0.92, 95% CI [0.90, 0.94]) and post-test (ICC = 0.94, 95% CI [0.92, 0.96]), supporting the reliability of the blinded dual-rating procedure. Mean attendance across the 12 weekly sessions was 94.2% in the AI group and 94.6% in the control group. A chi-square test of attendance (attended vs. missed sessions) by group indicated no significant difference, χ^2^(1) = 0.25, *p* = 0.616, indicating that the two conditions were matched on session exposure.

**Table 3:** Baseline characteristics by group.

Characteristic	Control (*n* = 152)	AI group (*n* = 155)	*p*
Age (years), M ± SD	19.43 ± 1.13	19.44 ± 1.14	0.885
Female, *n* (%)	97 (63.8%)	85 (54.8%)	—
Male, *n* (%)	55 (36.2%)	70 (45.2%)	—
Competence	3.01 ± 0.61	3.02 ± 0.55	0.887
Autonomy	3.00 ± 0.66	2.99 ± 0.59	0.893
Relatedness	2.93 ± 0.66	3.05 ± 0.69	0.120
Intrinsic motivation	3.14 ± 0.68	3.16 ± 0.65	0.828
Identified regulation	3.31 ± 0.62	3.30 ± 0.64	0.934
Introjected regulation	2.88 ± 0.53	2.86 ± 0.52	0.862
External regulation	3.13 ± 0.48	3.08 ± 0.56	0.367
RAI (composite)	3.78 ± 3.80	3.99 ± 3.89	0.632
Learning interest	3.06 ± 0.51	2.97 ± 0.48	0.094
Satisfaction	3.01 ± 0.55	3.03 ± 0.52	0.752
Engagement	2.99 ± 0.60	3.03 ± 0.58	0.615
Concentration	3.02 ± 0.60	2.99 ± 0.66	0.625
Vitality	2.98 ± 0.56	3.03 ± 0.56	0.445
Motor skill performance	2.80 ± 0.59	2.71 ± 0.60	0.212

RAI = Relative Autonomy Index. Values are M ± SD unless otherwise indicated. All baseline comparisons were nonsignificant (*p* > 0.05).

### 3.2 Intervention effects on need satisfaction, motivation, and motor skill

One-way ANCOVAs controlling for the corresponding pre-test score (Table 4) revealed that, at post-test, the AI feedback group scored significantly higher than the control group on competence, *F*(1, 304) = 9.28, *p* = 0.003, partial η^2^ = 0.030; autonomy, *F*(1, 304) = 10.61, *p* = 0.001, partial η^2^ = 0.034; and motor skill performance, *F*(1, 304) = 6.67, *p* = 0.010, partial η^2^ = 0.022. The composite relative autonomy index (RAI) also favored the AI group, *F*(1, 304) = 4.18, *p* = 0.042, partial η^2^ = 0.014, as did external regulation (in the expected direction, with the AI group reporting lower external regulation), *F*(1, 304) = 6.43, *p* = 0.012. In contrast, relatedness did not differ between groups, *F*(1, 304) = 0.001, *p* = 0.974. Among the secondary outcomes, learning interest, *F*(1, 304) = 8.82, *p* = 0.003, and engagement, *F*(1, 304) = 5.08, *p* = 0.025, favored the AI group, whereas satisfaction, concentration, and vitality did not differ significantly (Table 4).

**Table 4:** ANCOVA results: post-intervention outcomes controlling for baseline.

Outcome	*F*(1,304)	Partial η^2^	*p*	Adj. means (Control / AI)
Competence	9.28	0.030	0.003	3.039/3.234 *
Autonomy	10.61	0.034	0.001	2.970/3.166 *
Relatedness	0.001	<0.001	0.974	2.965/2.979 ns
RAI (composite)	4.18	0.014	0.042	3.592/4.548 *
Intrinsic motivation	1.43	0.005	0.234	3.118/3.206 ns
Identified regulation	0.66	0.002	0.417	3.316/3.374 ns
Introjected regulation	1.26	0.004	0.263	2.941/2.877 ns
External regulation	6.43	0.021	0.012	3.151/2.981 *
Learning interest	8.82	0.028	0.003	3.036/3.185 *
Satisfaction	1.31	0.004	0.254	3.004/3.080 ns
Engagement	5.08	0.016	0.025	2.949/3.095 *
Concentration	0.03	<0.001	0.857	3.019/3.024 ns
Vitality	2.10	0.007	0.148	2.984/3.082 ns
Motor skill performance	6.67	0.022	0.010	2.914/3.054 *

Adjusted means are estimated at the mean pre-test score. *p < 0.05; ns = not significant. Cohen’s *f*: 0.10 = small, 0.25 = medium, 0.40 = large.

These effects were corroborated by linear mixed models testing the Group × Time interaction, with random intercepts for class and participant (Table 5 and Figure 3). Significant Group × Time interactions emerged for competence, *b* = 0.185, SE = 0.092, *p* = 0.045, 95% CI [0.004, 0.366]; autonomy, *b* = 0.205, SE = 0.092, *p* = 0.025, 95% CI [0.026, 0.385]; motor skill performance, *b* = 0.225, SE = 0.081, *p* = 0.006, 95% CI [0.066, 0.384]; and learning interest, *b* = 0.244, SE = 0.072, *p* < 0.001, 95% CI [0.104, 0.385], indicating greater pre-to-post gains in the AI group. The Group × Time interaction for the composite RAI was not statistically significant in the mixed-model specification, *b* = 0.746, SE = 0.592, *p* = 0.209, and relatedness showed no significant interaction, *b* = −0.107, *p* = 0.274.

**Table 5:** Linear mixed models: Group × Time interaction effects (with class random effect).

**Outcome**	**Estimate**	**SE**	***p***	**95% CI**	**ICC_class**
Competence	0.185	0.092	0.045	[0.004, 0.366]	0.000
Autonomy	0.205	0.092	0.025	[0.026, 0.385]	0.000
Relatedness	−0.107	0.098	0.274	[−0.298, 0.084]	0.000
RAI (composite)	0.746	0.592	0.209	[−0.415, 1.906]	0.000
Intrinsic motivation	0.072	0.099	0.470	[−0.122, 0.265]	0.000
Identified regulation	0.064	0.101	0.523	[−0.133, 0.262]	0.000
Introjected regulation	−0.053	0.083	0.523	[−0.215, 0.109]	0.000
External regulation	−0.117	0.089	0.193	[−0.292, 0.058]	0.000
Learning interest	0.244	0.072	<0.001	[0.104, 0.385]	0.000
Satisfaction	0.057	0.078	0.466	[−0.096, 0.210]	0.004
Engagement	0.112	0.082	0.170	[−0.048, 0.272]	0.015
Concentration	0.039	0.089	0.660	[−0.136, 0.215]	0.026
Vitality	0.050	0.084	0.554	[−0.115, 0.215]	0.006
Motor skill performance	0.225	0.081	0.006	[0.066, 0.384]	0.000

Model: Outcome ~ Group × Time + (1|Class) + (1|ID). Estimate = coefficient of the Group × Time interaction. ICC(class) = proportion of variance at the class level. Degrees of freedom via Satterthwaite approximation.

**Figure 3:**
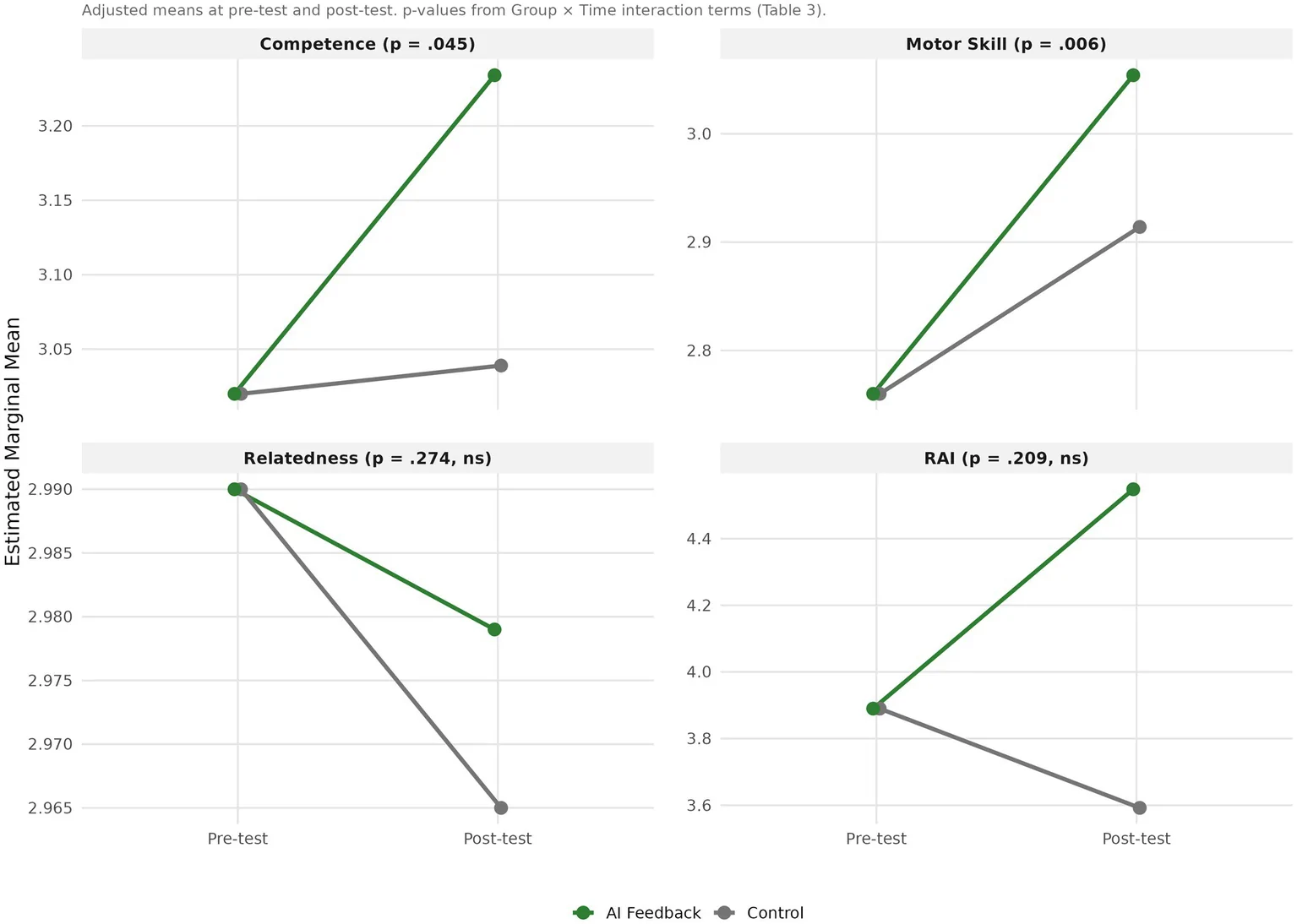
Group × Time interaction plots for **(a)** competence, **(b)** autonomy, **(c)** relatedness, and **(d)** relative autonomy index (RAI). Adjusted means are plotted at pre-test and post-test for the AI real-time feedback group and the control group. Error bars represent ±1 SE. Significance levels from linear mixed models: *p < 0.05, **p < 0.01, ***p < 0.001, ns = not significant.

Taken together, these analyses support Hypothesis 1: relative to the control condition, the AI feedback group showed greater gains in competence and motor skill, whereas relatedness remained unaffected as predicted. Support for the autonomous-motivation component of H1 was mixed—the group difference in RAI was significant in the covariate-adjusted ANCOVA but not in the mixed-model interaction test—suggesting a smaller and less robust effect on the composite motivation index than on competence and motor skill.

### 3.3 Mediation of intervention effects (structural equation model)

To test the hypothesized self-determination pathways (H2–H4), a structural equation model was estimated in lavaan with robust maximum likelihood estimation (MLR, Figure 4), specifying RAI as a latent factor with the four motivational subscales as indicators. The model fit the data well: χ^2^(30) = 44.65, *p* = 0.042; CFI = 0.940; TLI = 0.910; RMSEA = 0.040, 90% CI [0.008, 0.063]; SRMR = 0.044. All four subscales loaded significantly on the RAI latent factor in the expected directions (intrinsic *β* = 0.67, identified *β* = 0.61, introjected *β* = −0.41, external *β* = −0.59; all *p* < 0.001).

**Figure 4:**
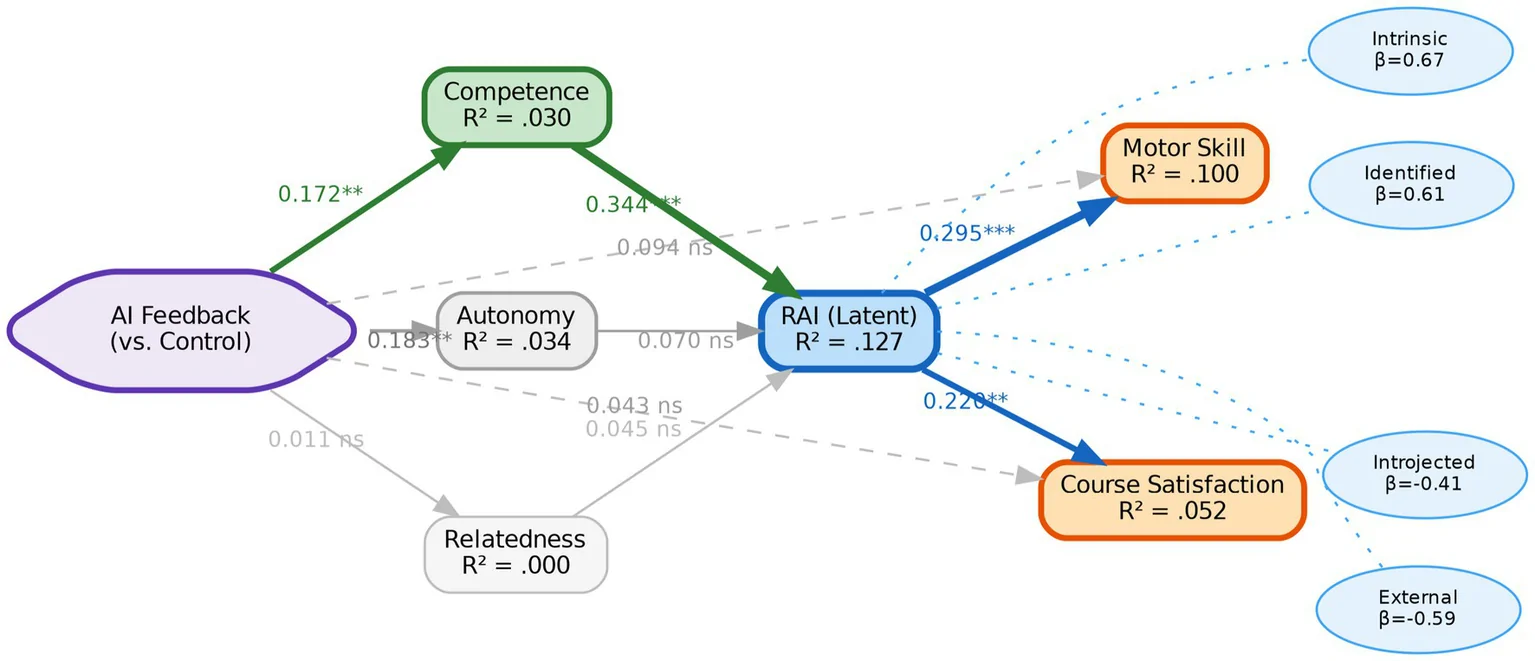
Path diagram of the structural equation model showing standardized path coefficients from the AI feedback group assignment through basic psychological needs and autonomous motivation to motor skill performance and course satisfaction. Significant paths (*p* < 0.05) are shown with solid lines; nonsignificant paths with dashed lines. *R*^2^ values indicate variance explained in each endogenous variable.

Consistent with H2, group assignment significantly predicted competence, *b* = 0.194, SE = 0.064, *p* = 0.002, *β* = 0.172, and competence in turn significantly predicted RAI, *b* = 0.259, SE = 0.047, *p* < 0.001, *β* = 0.344. Although group assignment also predicted autonomy, *b* = 0.196, *p* = 0.001, the autonomy-to-RAI path was nonsignificant, *b* = 0.056, *p* = 0.269. Thus, among the three needs, competence was the primary need linking AI feedback to autonomous motivation, supporting H2.

Supporting H3, the relatedness pathway was nonsignificant at both stages: group did not predict relatedness, *b* = 0.014, *p* = 0.842, and relatedness did not predict RAI, *b* = 0.032, *p* = 0.497. This pattern provides discriminant validity for the need-specificity prediction, indicating that the motivational benefit of AI feedback was carried specifically by competence rather than by relatedness.

Regarding H4, RAI significantly predicted both motor skill performance, *b* = 0.380, SE = 0.097, *p* < 0.001, *β* = 0.295, and course satisfaction, *b* = 0.290, SE = 0.088, *p* = 0.001, *β* = 0.220. The specific indirect effect of AI feedback on motor skill through the competence-to-RAI chain was significant, *b* = 0.019, SE = 0.008, 95% bias-corrected bootstrap CI [0.004, 0.038], as was the total indirect effect on motor skill, *b* = 0.023, SE = 0.010, 95% CI [0.004, 0.045]. Critically, the direct group-to-skill path was nonsignificant, *b* = 0.103, SE = 0.062, *p* = 0.097, indicating that the effect of AI feedback on motor skill was fully mediated by the competence-to-RAI sequence. For course satisfaction, the specific and total indirect effects were also significant (competence-to-RAI-to-satisfaction: *b* = 0.015, 95% CI [0.001, 0.034]; total indirect: *b* = 0.018, 95% CI [0.002, 0.039]), although the total effect of group on satisfaction did not reach significance, *b* = 0.066, *p* = 0.296. The model accounted for 10.0% of the variance in motor skill and 5.2% of the variance in satisfaction. These results support H4 for motor skill, demonstrating that the competence → RAI → performance mediation chain accounted for a substantial proportion of the AI feedback effect, and provide partial support for the satisfaction outcome, for which a significant indirect (but not total) effect was observed (Table 6).

**Table 6:** Structural equation model: path analysis with RAI latent factor.

**Path**	**Estimate**	**SE**	***p***	***β***
Competence ← Group	0.194	0.064	0.002	0.172
Autonomy ← Group	0.196	0.060	0.001	0.183
Relatedness ← Group	0.014	0.068	0.842	0.011
RAI ← Competence	0.259	0.047	<0.001	0.344
RAI ← Autonomy	0.056	0.050	0.269	0.070
RAI ← Relatedness	0.032	0.047	0.497	0.045
Skill ← RAI	0.380	0.097	<0.001	0.295
Skill ← Group	0.103	0.062	0.097	0.094
Satisfaction ← RAI	0.290	0.088	0.001	0.220
Satisfaction ← Group	0.048	0.063	0.448	0.043

Model fit: χ^2^(30) = 44.65, *p* = 0.042; CFI = 0.940; TLI = 0.910; RMSEA = 0.040 [0.008, 0.063]; SRMR = 0.044. RAI factor loadings: intrinsic *β* = 0.67; identified *β* = 0.61; introjected *β* = −0.41; external *β* = −0.59 (all *p* < 0.001).

Indirect and total effects:

**Table:** 

**Indirect / Total effect**	**Estimate**	**SE**	***p***
Competence → RAI → Skill	0.019	0.008	0.022
Competence → RAI → Satisfaction	0.015	0.007	0.031
Total indirect → Skill	0.023	0.010	0.023
Total indirect → Satisfaction	0.018	0.008	0.020
Total effect → Skill	0.126	0.062	0.040
Total effect → Satisfaction	0.066	0.063	0.296

Group coded 0 = control, 1 = AI feedback. β = standardized estimate. *R*^2^: Competence = 0.030; RAI = 0.127; Motor Skill = 0.100; Satisfaction = 0.052.

## 4 Discussion

The present study examined whether and how AI-mediated real-time feedback influences university students’ motivation and motor skill learning in a Zhuang Daoyin course, using self-determination theory (SDT) to specify the psychological pathways involved. Moving beyond the technology-to-outcome question of whether AI feedback improves performance, we tested a need-specific mediational account in which AI feedback was expected to operate through competence satisfaction, and thereby through autonomous motivation, rather than through an undifferentiated positive response. The findings largely supported this account: relative to traditional demonstration–imitation–verbal correction, AI feedback produced greater gains in competence, autonomous motivation, and motor skill, left relatedness unaffected, and exerted its effect on motor skill entirely through a competence-to-autonomy sequence.

### 4.1 AI real-time feedback enhances competence and motor skill

Consistent with Hypothesis 1, students in the AI feedback condition showed significantly greater improvement than the control group in competence, autonomous motivation, and motor skill performance, whereas relatedness remained unchanged. This pattern aligns with SDT’s contention that need-satisfying learning environments foster higher-quality motivation and, in turn, better learning outcomes (Cheon et al., 2012; Ntoumanis and Standage, 2009; Standage et al., 2003). It also converges with computer-assisted motor-learning research indicating that immediate, individualized, and visually explicit feedback supports error detection and movement correction (Hsia and Hwang, 2020; Huang et al., 2023; Zhu et al., 2020). Importantly, the present results extend that literature by showing that the benefit of such feedback is not confined to observable technical indicators but also registers in students’ psychological experience of the learning process—specifically, in a strengthened sense of competence. Directly relevant here, Hsia et al. (2025a) showed that an AI-supported physical skill auto-assessment and feedback system incorporating a reflection-promoting mechanism improved yoga skill, intrinsic motivation, and critical thinking relative to conventional teacher-led instruction, supporting the view that immediate AI feedback can enhance both performance and motivational experience in motor-skill learning.

The effect on motor skill was robust across both the covariate-adjusted ANCOVA and the mixed-model Group × Time interaction, and negligible class-level and teacher-level intraclass correlations indicated that these effects were not artifacts of clustering within intact classes or of particular instructors. Because instruction of new movements was standardized and delivered by the same three instructors across both conditions in a crossed design, the group difference is most plausibly attributable to the source of corrective feedback during practice—real-time algorithmic feedback versus post-hoc verbal correction—rather than to instructor effects or content differences.

### 4.2 Competence as the specific pathway: evidence for need-specificity

The mediation results provide the study’s central theoretical contribution. Supporting Hypothesis 2, competence was the only basic need that significantly linked AI feedback to autonomous motivation: group assignment predicted competence, and competence in turn predicted the relative autonomy index (RAI). Although group assignment also raised autonomy satisfaction, the autonomy-to-RAI path was nonsignificant, indicating that the motivational consequences of AI feedback were carried primarily by competence rather than autonomy. Consistent with this need-specificity, Hsia et al. (2025b) reported that an AI-facilitated peer-assisted learning system elevated all three SDT needs; yet, because that study compared conditions via ANCOVA without modeling the need-to-skill mediation path, it could not establish which need carried the effect. The present SEM addresses precisely this gap by showing that competence alone transmits the motivational benefit.

Supporting Hypothesis 3, the relatedness pathway was nonsignificant at both stages—AI feedback did not affect relatedness, and relatedness did not predict RAI. Rather than a limitation, this null pathway is theoretically informative. It provides discriminant evidence that the intervention operated through the functions it actually affords: real-time posture recognition makes progress legible and correction attainable, thereby supporting competence, but it contains no interpersonal component and therefore should not, and did not, strengthen relatedness, which depends on teacher and peer connection. This need-specific pattern advances SDT-based analyses of educational technology by showing that a tool’s motivational impact can be traced to its specific affordances rather than to a diffuse enthusiasm effect. It also refines the complete-mediation model reported by Wei et al. (2026b), in which responsibility-granting teaching engaged all three needs: whereas autonomy-supportive pedagogy can satisfy multiple needs simultaneously, a narrowly targeted technological affordance appears to engage a single need selectively.

### 4.3 A complete competence-to-motivation mediation of motor skill

Hypothesis 4 was supported for motor skill performance. The indirect effect of AI feedback on motor skill through the competence-to-RAI sequence was significant, and the direct group-to-skill path was nonsignificant, indicating full mediation: AI feedback improved motor skill because it enhanced competence satisfaction, which supported more autonomous motivation, which in turn predicted better performance. This complete-mediation structure parallels the pattern observed by Wei et al. (2026b) for autonomy-supportive teaching and extends it to a technological intervention, reinforcing the view that instructional tools in traditional ethnic sports education influence outcomes to the extent that they successfully engage students’ basic psychological needs.

These findings also extend our prior work with the same RTMPose-based system (Wei et al., 2026a). Whereas the earlier study explained the skill benefit through behavioral-cognitive mediators such as self-regulated learning and practice time, the present study isolates the psychological-need mechanism proposed by SDT, showing that competence satisfaction and the internalization of motivation constitute a distinct and theoretically specified route from AI feedback to performance. Taken together, the two studies suggest complementary behavioral and motivational pathways: AI feedback appears to increase both how much students practice and how autonomously they are motivated to do so, with competence as a common psychological anchor. Although the two studies drew on the same system, course, and institutional context, they were conducted with distinct participant samples and separately collected motor-skill outcome data, with no overlap in participants or outcomes between the two works.

For course satisfaction, the evidence was weaker. Although the specific and total indirect effects through competence and RAI were significant, the total effect of group on satisfaction was not, and the direct path was nonsignificant. Thus, AI feedback was associated with satisfaction only indirectly, via improved competence and autonomous motivation, and did not produce a detectable overall difference in how satisfied students were with the course. This is consistent with the possibility that course satisfaction is shaped by many factors beyond the feedback modality, including the instructor relationship and the broader course experience. One further qualification concerns the composite RAI. Although the ANCOVA detected a significant group difference in RAI (*p* = 0.042), this effect did not reach significance in the linear mixed model (*p* = 0.209), and the effect size was modest (η^2^p = 0.014). This pattern indicates that the motivational internalization process, while statistically detectable through the competence → RAI → outcome chain in SEM, did not produce a large mean-level shift in the composite motivation index over a single semester. A longer intervention window or more frequent sessions may be needed for competence gains to fully translate into stable shifts in motivational quality, consistent with developmental perspectives on internalization (Ryan and Deci, 2017).

### 4.4 Links with broader motor-learning and cognitive frameworks

The present findings connect with motor-learning perspectives in three respects. First, from the standpoint of the OPTIMAL theory of motor learning (Wulf and Lewthwaite, 2016), enhanced expectancies and autonomy support are two of the three key factors that facilitate learning. Real-time visual feedback that makes incremental improvement visible may function as a form of enhanced expectancy—the learner sees deviation shrinking in real time, which builds confidence in the ability to master the movement. Although we did not test this mechanism directly, the pattern in which competence (rather than autonomy) was the primary mediator is consistent with an expectancy-based account within the OPTIMAL framework. Second, real-time feedback may reduce extraneous cognitive load during the practice phase. In the control condition, students performing a complex movement sequence must simultaneously monitor their own body position, recall the teacher’s earlier demonstration, and wait for delayed verbal correction—a multi-demand situation that may strain working memory (Sweller et al., 2011). The AI overlay externalizes the comparison between performed and target postures, potentially offloading part of this monitoring burden and freeing cognitive resources for error correction. Wei et al. (2026a) observed a parallel mechanism involving self-regulated learning time, which is compatible with a cognitive-load interpretation: when students do not need to hold a mental image of the correct posture while simultaneously executing the movement, they may spend more time in focused, deliberate practice. Third, the null relatedness finding converges with meta-analytic evidence that basic psychological need effects in educational settings are partially domain- and context-specific. Whereas teacher–student relationships consistently emerge as a strong predictor of relatedness satisfaction (Roorda et al., 2011), technology-mediated environments that lack interpersonal interaction may produce smaller or null effects on relatedness unless explicit social features (e.g., collaborative learning tools, peer comparison dashboards) are integrated. The present findings suggest that real-time posture feedback is not a “social” technology in this sense—its motivational effect is competence-specific—and that future designs wishing to address the full SDT triad may need to add social feedback channels intentionally rather than expecting them to emerge from individual sensor-based feedback. A complementary contrast is provided by gamified AI feedback: Hsia et al. (2025c) found that a gamified intelligent tutoring system improved motor skill and learning engagement relative to a non-gamified AI feedback system. This suggests that the design layer layered onto real-time feedback—here, gamification versus plain posture visualization—shapes its motivational reach, and that our competence-specific result may be particular to non-gamified posture feedback rather than a general property of AI tutoring.

### 4.5 Limitations and future directions

Several limitations should be acknowledged. First, the study was conducted at a single university with second-year students in one traditional health-preservation course, which constrains generalizability to other institutions, age groups, and movement domains. Second, although allocation was randomized at the class level and clustering was explicitly modeled, the number of clusters (12 classes) was modest, and larger multi-site studies with more clusters would provide stronger tests of the class-level structure. Third, the 12-week window captures short-term acquisition but not retention or transfer. Consistent with the guidance hypothesis (Salmoni et al., 1984), high-frequency concurrent feedback can enhance immediate acquisition while its benefits fail to persist once feedback is withdrawn in retention or transfer conditions; longitudinal follow-up is needed to determine whether the motivational and skill gains persist and whether a novelty effect contributes to the early advantage. Fourth, the internal consistency of some composite indices was modest, and the motivational effect was method-dependent. This method-dependence (significant in ANCOVA, nonsignificant in LMM) suggests that the composite RAI effect, while directionally consistent, is sensitive to analysis strategy, and future studies should pre-register both the primary analytic method and the composite-scoring formula to reduce researcher degrees of freedom. Fifth, because the analysis relied on a latent-variable SEM that does not directly model multilevel clustering, we verified that class-level ICCs were low but recommend that future work employ multilevel SEM where cluster sizes permit. Sixth, a feedback-dosage asymmetry should be noted: the AI condition provided continuous, fully individualized concurrent feedback, whereas teacher-led terminal feedback could not match this per-student frequency across a full class, and no formal record of per-learner feedback frequency was kept in either arm. This asymmetry, which is intrinsic to the intervention rather than incidental to it, should be interpreted when comparing the two conditions, and future work should quantify feedback dosage in both arms.

Seventh, the interpretation of the null relatedness finding as discriminant evidence should be qualified. Without a formal equivalence test (e.g., TOST; Lakens et al., 2018), it cannot be statistically claimed that the AI and control groups were equivalent on relatedness—only that the null hypothesis was not rejected. Future replications should pre-register an equivalence margin (e.g., *d* = ±0.20) and conduct a TOST to strengthen the discriminant-validity argument.

Eighth, all psychological outcome variables were measured via self-report scales, which introduces potential common-method bias. Future studies should incorporate objective or third-party indicators (e.g., behavioral engagement metrics from system logs, peer-rated relatedness) to triangulate the self-report findings.

Ninth, the RTMPose system was trained on a general human pose dataset, and its accuracy may vary across body types, clothing styles, and skin tones that differ from the training distribution—a concern particularly relevant in ethnically diverse populations. Although the present sample was relatively homogeneous in age and body composition, future deployments of AI feedback in physical education should validate pose-estimation accuracy for the target population.

### 4.6 Practical implications and conclusion

The present findings carry three practical implications for the design and deployment of AI tools in traditional ethnic sports education. First, competence-support is a high-value design target. Systems that prioritize real-time, individual-level progress visualization—making deviation and improvement directly visible—are more likely to produce motivational benefits than those that focus narrowly on technical scoring or cumulative statistics. Second, the null effect on relatedness is not a design failure but a design constraint: posture-recognition AI lacks an interpersonal channel, and attempts to use such systems as all-in-one solutions risk disappointing both students and instructors. A division-of-labor model, in which AI provides scalable, precise competence feedback while teachers focus on relational and motivational support, is more promising than trying to replace human interaction with algorithmic feedback. Third, given the policy context of Healthy China 2030 and Education Informatization 2.0—and the shortage of qualified instructors in traditional ethnic sports—competence-oriented AI feedback may serve as a force multiplier, enabling teachers to preserve instructional quality even when class sizes are large or when specialist instructors are unavailable. Its value is not in substituting for the teacher but in augmenting the teacher’s capacity to provide individualized guidance at scale.

In conclusion, this study demonstrated that AI-mediated real-time posture feedback, relative to traditional instruction, improved competence satisfaction and motor skill performance in a Zhuang Daoyin physical education course, and that the skill benefit was substantially mediated by a competence → autonomous motivation → performance pathway. Relatedness was unaffected, providing discriminant evidence that the motivational benefit was functionally specific to competence rather than a diffuse positivity effect. These findings advance a need-specificity model of educational AI: the motivational value of a technology depends on which basic psychological needs its specific affordances can satisfy, and different needs require different design features. For traditional ethnic sports—carriers of cultural knowledge under pressure from modernization and instructor scarcity—AI tools designed with this principle in mind can serve not as replacements for human teaching but as targeted amplifiers of the learning experiences that matter most.

## Data Availability

The original contributions presented in the study are included in the article/Supplementary material, further inquiries can be directed to the corresponding author/s.

## References

[ref1] Campbell M. K. Piaggio G. Elbourne D. R. Altman D. G. (2012). CONSORT 2010 statement: extension to cluster randomised trials. BMJ 345, e5661–e5661. doi: 10.1136/bmj.e5661, 22951546

[ref2] Chen B. Vansteenkiste M. Beyers W. Boone L. Deci E. L. Van der Kaap-Deeder J. . (2015). Basic psychological need satisfaction, need frustration, and need strength across four cultures. Motiv. Emot. 39, 216–236. doi: 10.1007/s11031-014-9450-1

[ref3] Cheon S. H. Reeve J. Moon I. S. (2012). Experimentally based, longitudinally designed, teacher-focused intervention to help physical education teachers be more autonomy supportive toward their students. J. Sport Exerc. Psychol. 34, 365–396. doi: 10.1123/jsep.34.3.365 22691399

[ref4] Deci E. L. Ryan R. M. (2000). The “what” and “why” of goal pursuits: human needs and the self-determination of behavior. Psychol. Inq. 11, 227–268. doi: 10.1207/S15327965PLI1104_01

[ref5] Du H. Qin L. (2025). Understanding the black box: psychological mechanisms in AI-supported education. Br. J. Educ. Technol. 56, 412–428. doi: 10.1111/bjet.13456

[ref6] Enders C. K. Bandalos D. L. (2001). The relative performance of full information maximum likelihood estimation for missing data in structural equation models. Struct. Equ. Model. 8, 430–457. doi: 10.1207/S15328007SEM0803_5

[ref7] Faul F. Erdfelder E. Lang A. G. Buchner A. (2007). G*power 3: a flexible statistical power analysis program for the social, behavioral, and biomedical sciences. Behav. Res. Methods 39, 175–191. doi: 10.3758/BF03193146, 17695343

[ref8] Goudas M. Biddle S. Fox K. (1994). Perceived locus of causality, goal orientations, and perceived competence in school physical education classes. Br. J. Educ. Psychol. 64, 453–463. doi: 10.1111/j.2044-8279.1994.tb01116.x 7811633

[ref9] Grolnick W. S. Ryan R. M. (1989). Parent styles associated with children’s self-regulation and competence in school. J. Educ. Psychol. 81, 143–154. doi: 10.1037/0022-0663.81.2.143

[ref10] Guay F. Vallerand R. J. Blanchard C. (2000). On the assessment of situational intrinsic and extrinsic motivation: the situational motivation scale (SIMS). Motiv. Emot. 24, 175–213. doi: 10.1023/A:1005614228250

[ref11] Hox J. J. Moerbeek M. van de Schoot R. (2017). Multilevel Analysis: Techniques and Applications. 3rd Edn New York: Routledge.

[ref12] Hsia L. H. Hwang G. J. (2020). Enhancing students’ reflective thinking and movement skill performance in physical education through a mobile learning system. Br. J. Educ. Technol. 51, 1745–1763. doi: 10.1111/bjet.12988

[ref13] Hsia L. H. Hwang G. J. Lin Y. N. (2024). Fostering motor skill performance and self-regulated learning in physical education through AI-supported feedback. Comput. Educ. 220:105116. doi: 10.1016/j.compedu.2024.105116

[ref14] Hsia L. H. Hwang G. J. Lin Y. N. Hwang J. P. (2025a). Artificial intelligence-supported physical education during the pandemic: a physical skill auto-assessment and feedback approach based on a reflection-promoting mechanism. Educ. Technol. Res. Dev. 73, 1429–1450. doi: 10.1007/s11423-025-10452-7

[ref15] Hsia L. H. Lin Y. N. Lin C. H. Hwang G. J. (2025b). Prompting somatic practice performance with AI-facilitated peer-assisted learning: a self-determination theory perspective. Educ. Inf. Technol. 30, 23331–23364. doi: 10.1007/s10639-025-13681-8

[ref16] Hsia L. H. Lin Y. N. Lin C. H. Hwang G. J. (2025c). Effectiveness of gamified intelligent tutoring in physical education through the lens of self-determination theory. Comput. Educ. 227:105212. doi: 10.1016/j.compedu.2024.105212

[ref17] Hu L. Bentler P. M. (1999). Cutoff criteria for fit indexes in covariance structure analysis: conventional criteria versus new alternatives. Struct. Equ. Model. 6, 1–55. doi: 10.1080/10705519909540118

[ref18] Huang Y. Liu J. Chen S. (2023). Immediate visual feedback improves movement accuracy in novice learners: a systematic review. J. Sports Sci. 41, 712–724. doi: 10.1080/02640414.2023.2212345

[ref19] Jiang T. Lu P. Zhang L. Ma N. Han R. Lyu C. (2023). RTMPose: real-time multi-person pose estimation based on MMPose. arXiv preprint arXiv:2303.07399. doi: 10.48550/arXiv.2303.07399

[ref20] Jiang S. Wang L. Zhang T. (2024). Application of artificial intelligence in physical education: a scoping review. Comput. Educ. 210:104978. doi: 10.1016/j.compedu.2023.104978

[ref21] Kish L. (1965). Survey Sampling. New York: Wiley.

[ref22] Koo T. K. Li M. Y. (2016). A guideline of selecting and reporting intraclass correlation coefficients for reliability research. J. Chiropr. Med. 15, 155–163. doi: 10.1016/j.jcm.2016.02.012, 27330520 PMC4913118

[ref23] Lakens D. Scheel A. M. Isager P. M. (2018). Equivalence testing for psychological research: a tutorial. Adv. Methods Pract. Psychol. Sci. 1, 259–269. doi: 10.1177/2515245918770963

[ref24] Lazowski R. A. Hulleman C. S. (2016). Motivation interventions in education: a meta-analytic review. Rev. Educ. Res. 86, 602–640. doi: 10.3102/0034654315617832

[ref25] Li X. Chen Y. Zhao H. (2025). Artificial intelligence-driven personalized physical education: current status and prospects. Front. Psychol. 16:1423567. doi: 10.3389/fpsyg.2025.1423567

[ref26] Lin Y. N. Hsia L. H. Hwang G. J. (2022). Promoting pre-service teachers’ reflection and motor skill learning in physical education using a video annotation system. Interact. Learn. Environ. 30, 984–998. doi: 10.1080/10494820.2019.1703010, 37339054

[ref9001] McAuley E. Duncan T. Tammen V. V. (1989). Psychometric properties of the Intrinsic Motivation Inventory in a competitive sport setting: A confirmatory factor analysis. Research Quarterly for Exercise and Sport, 60, 48–58. doi: 10.1080/02701367.1989.10607413, 2489825

[ref27] Ministry of Education of the People’s Republic of China. (2018). Education Informatization 2.0 Action Plan. Beijing: Ministry of Education of the People’s Republic of China. Available online at: https://www.moe.gov.cn/srcsite/A16/s3342/201804/t20180425_334188.html (Accessed July 28, 2026).

[ref9002] Mo M. Lei S. Guo L. (2026). The inheritance and transcendence of China’s traditional ethnic sports in the digitalization process. Sport Science and Technology, 47, 55–61. doi: 10.14038/j.cnki.tykj.2026.05.014

[ref28] Muthén B. O. (1994). Multilevel covariance structure analysis. Sociol. Methods Res. 22, 376–398. doi: 10.1177/0049124194022003006

[ref29] Ntoumanis N. Standage M. (2009). Motivation in physical education classes: a self-determination theory perspective. Theory Res. Educ. 7, 194–202. doi: 10.1177/1477878509104324

[ref30] Preacher K. J. Hayes A. F. (2008). Asymptotic and resampling strategies for assessing and comparing indirect effects in multiple mediator models. Behav. Res. Methods 40, 879–891. doi: 10.3758/BRM.40.3.879, 18697684

[ref31] Roorda D. L. Koomen H. M. Y. Spilt J. L. Oort F. J. (2011). The influence of affective teacher–student relationships on students’ school engagement and achievement: a meta-analytic approach. Rev. Educ. Res. 81, 493–529. doi: 10.3102/0034654311421793

[ref32] Ryan R. M. Connell J. P. (1989). Perceived locus of causality and internalization: examining reasons for acting in two domains. J. Pers. Soc. Psychol. 57, 749–761. doi: 10.1037/0022-3514.57.5.749, 2810024

[ref33] Ryan R. M. Deci E. L. (2000). Self-determination theory and the facilitation of intrinsic motivation, social development, and well-being. Am. Psychol. 55, 68–78. doi: 10.1037/0003-066X.55.1.68, 11392867

[ref34] Ryan R. M. Deci E. L. (2017). Self-Determination Theory: Basic Psychological Needs in Motivation, Development, and Wellness. New York: Guilford Press.

[ref35] Ryan R. M. Frederick C. (1997). On energy, personality, and health: subjective vitality as a dynamic reflection of well-being. J. Pers. 65, 529–565. doi: 10.1111/j.1467-6494.1997.tb00326.x, 9327588

[ref9003] Ryan R. M. (1982). Control and information in the intrapersonal sphere: An extension of cognitive evaluation theory. Journal of Personality and Social Psychology, 43, 450–461. doi: 10.1037/0022-3514.43.3.450

[ref36] Salmoni A. W. Schmidt R. A. Walter C. B. (1984). Knowledge of results and motor learning: a review and critical reappraisal. Psychol. Bull. 95, 355–386. doi: 10.1037/0033-2909.95.3.355, 6399752

[ref9004] Schaufeli W. B. Martínez I. M. Pinto A. M. Salanova M. Bakker A. B. (2002). Burnout and engagement in university students: A cross-national study. Journal of Cross-Cultural Psychology, 33, 464–481. doi: 10.1177/0022022102033005003

[ref37] Shi Q. (2010). Development of the college student physical education learning satisfaction scale. J. Phys. Educ. 17, 56–60. doi: 10.16237/j.cnki.cn44-1404/g8.2010.07.028

[ref38] Standage M. Duda J. L. Ntoumanis N. (2003). A model of contextual motivation in physical education: using constructs from self-determination and achievement goal theories to predict physical activity intentions. J. Educ. Psychol. 95, 97–110. doi: 10.1037/0022-0663.95.1.97

[ref39] Sweller J. Ayres P. Kalyuga S. (2011). Cognitive Load Theory. New York: Springer.

[ref40] The State Council of the People’s Republic of China. (2016). Outline of the Healthy China 2030 Plan. Beijing: The State Council of the People’s Republic of China. Available at: https://www.gov.cn/zhengce/2016-10/25/content_5124174.htm (Accessed July 28, 2026).

[ref41] Vallerand R. J. (1997). “Toward a hierarchical model of intrinsic and extrinsic motivation,” in Advances in Experimental Social Psychology, ed. Zanna M. P., vol. 29 (San Diego: Academic Press), 271–360.

[ref42] Verbeke G. Molenberghs G. (2000). Linear Mixed Models for Longitudinal Data. New York: Springer.

[ref43] Wang J. Wang F. (2024). Application and challenges of artificial intelligence in physical education: a systematic review. Educ. Inf. Technol. 29, 12345–12370. doi: 10.1007/s10639-024-12578-y

[ref44] Wang J. Yu C. (2019). Development and norm establishment of the basic psychological needs satisfaction scale for adolescent sport and exercise. J. Xi’an Inst. Physical Educ. 36, 333–341,378. doi: 10.16063/j.cnki.issn1001-747x.2019.03.013

[ref45] Wei D. Yang Y. Chen G. Qin Y. Li R. (2026b). Pathways from responsibility granting to course satisfaction in university traditional health-preservation PE: a structural equation model based on self-determination theory. Sport Sci. Technol. 47, 145–151. doi: 10.14038/j.cnki.tykj.2026.03.035

[ref46] Wei D. Yang Y. Chen G. Wu G. Li R. Zhao C. (2026a). Construction and practice of a real-time feedback system for Zhuang Daoyin based on posture recognition. Sport Sci. Technol. 47, 40–49. doi: 10.14038/j.cnki.tykj.2026.04.009

[ref47] World Medical Association (2013). World medical association declaration of Helsinki: ethical principles for medical research involving human subjects. JAMA 310, 2191–2194. doi: 10.1001/jama.2013.281053 24141714

[ref48] Wulf G. Lewthwaite R. (2016). Optimizing performance through intrinsic motivation and attention for learning: the OPTIMAL theory of motor learning. Psychon. Bull. Rev. 23, 1382–1414. doi: 10.3758/s13423-015-0999-9, 26833314

[ref50] Zhu X. Ennis C. D. Chen A. (2020). Effects of augmented feedback on motor skill learning in physical education: a meta-analysis. Res. Q. Exerc. Sport 91, 459–473. doi: 10.1080/02701367.2019.1680156

