# Supplementary material for: Effects of AI-mediated real-time feedback on motivation and motor skill learning in traditional ethnic sports education: testing self-determination theory mechanisms
Source: Front Psychol. 2026 Oct 16;17:1958348. doi: 10.3389/fpsyg.2026.1958348 (PMC13720675; doi:10.3389/fpsyg.2026.1958348)
Supplement: Supplementary file 1 [file Data_Sheet_1.PDF]

声明：本伦理委员会组成和工作程序符合 GCP 原则和国家相关法律法规

批准证明书

广西中医药大学科技伦理委员会 GXTCMU-EC
广西中医药大学　　广西南宁市五合大道13号　　5301000
主任委员/副主任委员：肖健　　联系 电话：0771-3137535

批准号：GXTCMU-EC KS20251210-27
批件有效期： 2025 年 0 7 月 10 日-2027 年 0 7 日 30 日
项目名称：基于“壮族导引术”的高校公共体育课程改革研究与实践
项目负责人：韦东谊
主要研究者：韦东谊　　　　　电话：13977164677
所在单位：广西中医药大学体育部
研究负责机构：广西中医药大学
审查文件：

1、伦理审查申请表
2、科研项目登记信息
3、研究方案
4、临床研究保密承诺
5、主要研究者履历表
6、研究经济利益声明

审查决定：

“基于“壮族导引术”的高校公共体育课程改革研究与实践”的上述文件，经审查讨论符合伦理要求，批准本项目开展。

请研究者按照 GCP 以及我国相关法规要求执行本项目，相关要求如下：

一、若项目在批准证明书有效期内未启动，则批件自动失效。若研究需继续进行，请应在有效期满前 2 个月通过“研究进展报告”或“跟踪审查申请”递交延长申请，由广西中医药大学科技伦理委员会审核决定是否延长及延长期限。

二、因实施方案调整、外部环境变化等可能导致科技伦理风险发生变化的，项目负责人应及时报告学校科技伦理委员会进行评估，提出继续实施、暂停实施等意见，必要时，重新开展伦理审查，获得批准后执行。

三、完成本研究，请提交结题报告。暂停/提前终止/完成的科研项目，请及时通知本伦理委员会。

广西中医药大学涉人伦理委员会
2025 年 0 7 月 10 日

Ethics Approval Certificate

Statement: The composition and working procedures of this Ethics Committee comply with GCP principles and relevant national laws and regulations.

Approval Certificate

Science and Technology Ethics Committee of Guangxi University of Chinese Medicine (GXTCMU-EC)

Address: No. 13 Wuhe Avenue, Nanning, Guangxi, 530100, China

Chairperson/Vice-Chairperson: Xiao Jian
Telephone: 0771-3137535C

Approval Number: GXTCMU-EC-KS20251210-27

Validity Period of Approval: July 10, 2025 – July 30, 2027

Project Title: Reform and Practice of University Public Physical Education Curriculum Based on "Zhuang Daoyin Exercises"

Project Leader: Wei Dongyi

Principal Investigator: Wei Dongyi
Telephone: 13977164677

Affiliation: Department of Physical Education, Guangxi University of Chinese Medicine

Research Host Institution: Guangxi University of Chinese Medicine

Documents Reviewed:

1.Ethics Review Application Form
2.Research Project Registration Information
3.Research Protocol
4.Clinical Research Confidentiality Commitment
5.Curriculum Vitae of the Principal Investigator
6.Research Financial Interest Declaration

Review Decision:

The above documents for the project titled "Reform and Practice of University Public Physical Education Curriculum Based on 'Zhuang Daoyin Exercises'" have been reviewed and discussed. The project is determined to comply with ethical requirements and is **hereby approved** for implementation.

Please Note:

The investigators are required to conduct this project in accordance with GCP and relevant Chinese laws and regulations. The following conditions apply:

1. If the project has not been initiated within the validity period of this approval certificate, the approval shall automatically become invalid. If the study needs to be continued, an extension application should be submitted via a "Research Progress Report" or "Follow-up Review Application" **two months prior to the expiration date**. The extension decision and duration will be determined by the Science and Technology Ethics Committee of Guangxi University of Chinese Medicine upon review.

2. If adjustments to the implementation plan, changes in the external environment, or other factors may lead to changes in scientific and technological ethical risks, the project leader shall promptly report to the University's Science and Technology Ethics Committee for reassessment. The Committee may recommend continuation, suspension, or other actions, and if necessary, a new ethics review shall be conducted, and the project may only proceed after obtaining renewed approval.

3. Upon completion of this study, a final report shall be submitted. For research projects that are suspended, terminated early, or completed, the Ethics Committee shall be notified in a timely manner.
