# Supplementary material for: Effects of AI-mediated real-time feedback on motivation and motor skill learning in traditional ethnic sports education: testing self-determination theory mechanisms
Source: Front Psychol. 2026 Oct 16;17:1958348. doi: 10.3389/fpsyg.2026.1958348 (PMC13720675; doi:10.3389/fpsyg.2026.1958348)
Supplement: Supplementary file 2 [file Data_Sheet_2.PDF]

Supplementary Material: English Abstracts of Two Reference Articles (Wei et al., 2026a, 2026b)

The following English abstracts are provided as supplementary material to support the reviewer verification of two references cited in the manuscript. Both articles are published in the peer-reviewed Chinese journal Sport Science and Technology (《体育科技》). Digital Object Identifiers (DOIs) and CNKI online-first links are provided below for immediate verification.

Wei et al. (2026a)

Wei, D., Yang, Y., Chen, G., Wu, G., Li, R., and Zhao, C. (2026a). Construction and practice of a real-time feedback system for Zhuang Daoyin based on posture recognition. Sport Science and Technology 47, 40–49.

DOI: 10.14038/j.cnki.tykj.2026.04.009
CNKI online-first: https://link.cnki.net/urlid/45.1171.G8.20260630.1109.018
Published online: 2026-06-30

To address the difficulty of skill acquisition in online physical education caused by the absence of real-time feedback, the authors developed and validated an artificial-intelligence real-time feedback system based on posture recognition. Using a 10-week pre-test/post-test quasi-experimental design in a university Zhuang Daoyin "He Xu Le" course, 306 students were divided into an experimental group (AI real-time feedback) and a control group (traditional online platform) for comparison. The results showed that the system significantly improved students' movement quality, performance level, learning interest, and self-directed learning ability. Mediation analyses indicated that the system fully mediated the skill-improvement effect through enhanced self-directed learning ability and extended practice time. Qualitative interviews further confirmed that AI feedback was of core value in providing precise guidance and stimulating practice motivation, while also revealing limitations such as insufficient emotional support and limited recognition accuracy. The study demonstrates that the AI real-time feedback system can effectively optimize online motor-skill learning through a "cognitive-load reduction—motivation activation—behavioral engagement" pathway. It is recommended that future online physical education adopt a human–AI collaborative blended-teaching model, in which the AI system undertakes scalable, precise feedback tasks while teachers focus on higher-order guidance and humanistic motivation, jointly building an efficient and warm intelligent physical-education learning ecosystem.

Keywords: posture recognition; real-time feedback; online physical education; Zhuang Daoyin "He Xu Le"; learning motivation; self-directed learning ability

Wei et al. (2026b)

Wei, D., Yang, Y., Chen, G., Qin, Y., and Li, R. (2026b). Pathways from responsibility granting to course satisfaction in university traditional health-preservation PE: a structural equation model based on self-determination theory. Sport Science and Technology 47, 145–151.

DOI: 10.14038/j.cnki.tykj.2026.03.035
CNKI online-first: https://link.cnki.net/urlid/45.1171.G8.20260429.1633.070
Published online: 2026-04-30

Grounded in self-determination theory, this study constructed a structural equation model to examine the mechanism by which responsibility granting in course evaluation influences students' satisfaction with a traditional health-preservation physical education (PE) course. Using a questionnaire survey, the authors administered scales measuring perceived responsibility, basic psychological need satisfaction, motivational level, and course satisfaction to 343 first-year students from five universities of Chinese medicine. The results showed that the model fit the observed data well. The study found that granting students greater responsibility during course evaluation helped satisfy their basic psychological needs, thereby promoting the development of autonomous motivation. Specifically, responsibility granting significantly improved students' overall satisfaction with the traditional health-preservation PE course by enhancing autonomous motivation through the satisfaction of the needs for competence, autonomy, and relatedness (a complete-mediation model), providing a theoretical basis for the reform of the PE course evaluation system.

Keywords: self-determination theory; responsibility granting; basic psychological needs; autonomous motivation; course satisfaction; traditional health-preservation physical education
