# Supplementary material for: Effects of AI-mediated real-time feedback on motivation and motor skill learning in traditional ethnic sports education: testing self-determination theory mechanisms
Source: Front Psychol. 2026 Oct 16;17:1958348. doi: 10.3389/fpsyg.2026.1958348 (PMC13720675; doi:10.3389/fpsyg.2026.1958348)
Supplement: Supplementary file 3 [file Data_Sheet_3.PDF]

DOI：10.14038/j.cnki.tykj.2026.04.009　网络首发时间：2026-06-30 14:46:27　网络首发地址：https://link.cnki.net/urlid/45.1171.G8.20260630.1109.018

基于姿态识别的壮族导引实时反馈系统构建与实践

韦东谊[1]　杨　宇[2]　陈桂真[1]　吴国天[1]　李荣源[1]　赵长军[1]

（1.广西中医药大学体育部，广西 南宁 530200；2.广西中医药大学第一附属医院，广西 南宁 530001）

摘要：为解决线上体育教育因缺乏实时反馈导致的技能掌握困难，作者开发并验证了一套基于姿态识别的人工智能实时反馈系统。研究采用为期 10 周的前后测准实验设计，在某大学壮族导引 · 和嘘勒课程中，将 306 名学生分为实验组（AI 实时反馈）与对照组（传统学习通平台）进行对比分析。结果显示，该系统显著提升了学生的动作质量、演练水平、学习兴趣及自主学习能力。中介效应分析表明，系统通过增强自主学习能力与延长学习时长完全中介了技能提升效应。质性访谈进一步证实，AI 反馈在提供精准指导和激发练习动力方面具有核心价值，同时也存在情感支持不足与识别精度有限等局限。研究表明，人工智能实时反馈系统能够通过“认知减负—动机激发—行为投入”路径有效优化在线运动技能学习。建议未来在线体育教育采用人机协同的混合教学模式：AI 系统承担可规模化的精准反馈任务，教师则专注于高阶指导与人文激励，共同构建高效且富有温度的智能体育学习生态。

关键词：姿态识别；实时反馈；线上体育教学；壮族导引 · 和嘘勒；学习积极性；自主学习能力

尽管在线教育的全国推广对促进教育公平起到了积极作用，但在体育教育这类高度依赖身体实践的领域中，这一目标尚未得到充分实现。与以知识传授为主的学科不同，体育教育强调复杂运动技能的掌握，这一过程依赖于持续的实践练习以及教师提供的个性化反馈与纠正。传统的线上体育教育通常依赖单向视频演示（如 MOOC），难以展现关键的互动机制。这导致学习者陷入“练习盲区”，无法准确评价自身演练水准和及时纠正错误。这种缺乏互动的教学方式不仅阻碍了技能的发展，也削弱了学习者的动机，容易引发被动模仿而非主动反思的学习行为[1]。

线上体育教学领域中，混合式学习、翻转课堂等创新模式虽已在实践探索中斩获初步成果，但其在可扩展的实时运动技能指导方面仍存在明显短板，尚未形成有效的解决路径，这一问题已成为制约其深化发展的关键挑战。而人工智能在教育领域的快速渗透与应用，正为这一核心难题提供更为直接的破解思路与解决方案。凭借姿态识别这一关键技术支撑，人工智能系统已可复制专家指导的核心功能，包括对学习者运动状态的自动化评价与针对性个性化反馈[2]。但现有研究多聚焦于技术实现细节，往往弱化了教育学与心理学在运动学习中的关键调控作用，导致 AI 反馈机制的设计与优化缺乏理论根基。为弥补这一不足，本研究旨在构建整合性理论框架，核心观点为：人工智能驱动的反馈机制需立足于运动学习理论、认知负荷理论（CLT）以及自我决定理论（SDT）三个互补视角，方能得到更全面的理解与针对性优化。

该整合性理论框架在壮族导引・和嘘勒的教学实践中更能凸显其价值。作为以壮医学理论为指导、基于花山壁画遗存挖掘整理而成的传统保健体育运动，壮族导引・和嘘勒兼具鲜明的壮族文化特色与独特的运动属性[3]—其动作柔和舒缓、讲究内外兼修，且对动作精准度要求极高，学习者仅通过视频模仿极易出现细节偏差，难以真正掌握动作精髓。从运动学习理论视角出发，依托该框架设计的人工智能系统，能够针对学习者的实时动作姿态提供即时、具象的反馈，有效帮助学习者快速校准动作偏差、优化姿势细节，从而突破纯视频教学的局限。从认知科学视角来看，此类自动化指导方式的核心价值在于降低学习者的额外认知负荷：无需学习者在完成动作的同时兼顾自我诊断与纠错，其心理资源得以从“双重任务”中解放，进而更专注于运动技能的关键细节掌握与深度内化。此外，从动机激发逻辑来看，这一机制对壮族导引・和嘘勒的学习尤为关键：该运动对动作精准度的高要求易使初学者因反复失误丧失信心，而人工智能系统可通过实时修正提升任务成功率，帮助学习者建立并强化胜任感；同时，其打破时空限制的灵活练习机

基金项目：广西壮族自治区高等教育本科教学改革工程项目“‘壮族导引术’融入高校公共体育课程的研究与实践”（项目编号：2025JGB258）。

第一作者简介：韦东谊（1973—）,学士，副教授，研究方向为传统保健体育、人工智能与运用。

会，让学习者能自主安排练习强度与节奏，充分保障学习自主性。这种 “需求满足—动机激发”的路径，与自我决定理论（SDT）所阐释的内在动机形成机制高度一致。

在上述多维度整合理论框架的指导下，本研究进一步设计并开展了对基于 RTMPose 技术的人工智能实时反馈系统的评估，该系统旨在为线上壮族导引・和嘘勒课程的教学与技能训练提供针对性支持。为精准验证系统效能，聚焦以下核心研究问题展开探究：

研究问题 1：相较于以视频推送、线上答疑为核心的学习通传统教学方式，基于人工智能的实时反馈系统是否能更显著地提升学习者在壮族导引・和嘘勒中的运动技能表现?

研究问题 2:相较于以“视频学习+异步互动”为核心的学习通传统网络课程模式，人工智能驱动的实时反馈系统在激发学生的内在学习兴趣、促进自主学习行为方面，是否具有更显著的成效?

研究问题 3：在壮族导引・和嘘勒的技能习得过程中，人工智能系统是通过何种中介机制促进运动技能提升的?

研究问题 4：学生对人工智能辅助下的线上壮族导引・和嘘勒教学环境的学习体验如何?

通过对上述问题的系统性探究与解答，本研究旨在达成三重目标：其一，为姿态识别技术在线上体育技能学习评估中的实践应用提供坚实的实证支撑；其二，结合运动学习、认知负荷及自我决定理论，为智能反馈系统作用于技能习得的内在机理夯实理论基础；最终为未来民族传统体育项目的智能学习环境设计、技术与教育理论的深度融合提供兼具理论价值与实践参考的启示。

1 文献综述

1.1 线上体育教学研究

新冠疫情的暴发与蔓延加速了线上体育教学的推广进程，而围绕其教学成效的相关研究也呈现出鲜明的双重效应。一方面，多项研究已证实其在同步教学、混合教学等不同模式下的应用有效性—数据表明，线上体育教育对学生的学习态度、身体活动参与度、体能水平及学习积极性等关键维度均产生了积极影响。另一方面，学界也逐渐形成共识，明确指出线上体育教育存在显著的教学局限：由于过度依赖预录视频等单向输出内容，学习过程往往缺乏即时互动性与社交联结，这不仅削弱学生的深度参与感，更成为制约实际运动技能精准掌握与有效提升的核心瓶颈[4]。

Ovens 等人（2022）的研究指出，线上体育教育当前面临的核心挑战在于需推动根本性教学改革——其关键并非简单调整形式，而是要超越传统以知识传递为核心的教学模式[5]。该研究进一步揭示，这一改革需求的根源在于线上体育教育普遍存在的“反馈困境”：标准线上体育教学模型因受限于单向输出特征，本质上难以向学习者提供及时、具体且具备纠偏价值的运动指导，而这类指导恰是运动技能精准习得的核心要素。更为关键的是，这种“反馈缺失”直接导致运动学习中至关重要的“感知（动作状态）—动作（执行调整）—反馈（效果评价）”闭环无法形成，这也构成了制约该教学模式深化发展的主要障碍。因此，有效弥补这一反馈缺口，已成为推动在线体育教育向高质量、可持续方向发展的核心前提。

1.2 人工智能反馈系统在运动技能学习中的应用

为破解在线体育教育中“反馈不足”这一核心瓶颈，人工智能技术的应用正成为学界研究的焦点。事实上，其在优化个性化学习体验、提升教学效能方面的潜力，已在多个学科领域得到充分验证。具体到运动技能学习这一细分领域，随着计算机视觉技术的迭代，基于姿态识别技术的人工智能系统已成为突破反馈困境的关键支撑：它能够通过实时捕捉、解析学习者的动作轨迹与姿态细节，精准生成以往线上教学中稀缺的“即时化、个性化、纠偏性”反馈。已有大量实证研究显示，此类系统在涵盖瑜伽、太极拳、大学公共体育课程等多种运动场景中，均能显著提升学习者的动作精准度与规范性，同时对身体素质改善、学习积极性激发及自主练习意识培养产生积极影响[6]。

然而，尽管现有研究证实了技术应用的积极成效，但对相关文献的批判性梳理表明，该领域在理论体系构建与研究方法论设计层面仍存在显著短板。当前研究普遍呈现“技术导向”与“结果导向”的倾向：多聚焦于姿态识别算法的优化、系统功能的实现，以及动作准确性、学习积极性等显性绩效成果的验证，却往往将“反馈输入—技能习得”之间的学习过程视为“黑箱”——即对技术作用于学习效果的内在机理缺乏深入探究[7]。由此，一系列基础性理论问题尚未得到系统性解答：人工智能驱动的实时反馈机制究竟通过何种路径影响运动技能习得？其对学习者的认知加工过程与动机心理状态存在怎样的具体作用机制？这种核心理论基础的缺失，不仅导致相关智能系统的迭代优化缺乏科学依据，更极大

削弱了研究成果的可推广性。因此，该领域研究重心从单纯的技术开发与效果验证，转向理论驱动的机制探究，唯有厘清技术作用于学习的底层逻辑，才能真正释放人工智能在体育教育中的教学潜力。

1.3 理论框架

为回应上述研究缺口、突破“黑箱”式分析的局限，本研究以一个整合性系统理论框架为核心指导。通过有机融合三种具有互补性的理论视角，本研究不仅系统阐释了人工智能生成的实时反馈如何以及为何影响技能习得过程，构建了一条涵盖行为、认知与动机三个递进层面的因果作用链条。

该框架以运动学习理论为支撑，明确技能习得的行为基础。壮族导引・和嘘勒作为复杂且重形式的技能，其学习效果高度依赖对动作模式质量的准确把握，而这必须通过表现知识（KP）反馈实现—相较于仅反馈“结果好坏”的结果知识[8]（KR），KP 能提供动作细节层面的具体信息，对这类技能的掌握更为关键。为此，本研究设计的 AI 系统以供给关键 KP 信息为核心目标，旨在成为即时、高保真的纠正与指导载体，精准匹配运动学习理论对复杂技能习得的要求。

然而，KP 反馈呈现与传递需具备认知适配性（避免过量认知负担），这引出本框架的第二个理论支柱—认知负荷理论（CLT）。传统线上体育学习环境（如学习通）中，因缺乏自动化反馈，学习者需在模仿动作时额外承担“识别偏差、诊断错误”的认知任务，既分散对壮族导引・和嘘勒姿势、节奏等核心技能的注意力，又占用工作记忆，最终形成高外在认知负荷。而本研究的 AI 系统通过自动化“动作错误诊断”，承担部分非核心认知任务，成为“认知卸载”工具，可显著降低外在认知负荷。由此，学习者的工作记忆能更集中于“理解动作原理、修正细节、内化技能”等核心任务，最终促进这类复杂技能的深层理解与内化。

认知层面高效的学习过程，还需依托强有力的激励机制。为此，本研究框架以自我决定理论（SDT）为支撑——其核心观点是：内在动机的激发，关键在于能力、自主性等基本心理需求的满足[9]。本研究的 AI 系统设计即旨在直接满足这些需求：一方面通过提供清晰的技能掌握路径，强化学习者的能力感；另一方面通过赋予学习者对学习过程的一定控制权，保障其自主性。而基本心理需求的满足，能有效激发高质量内在动机，进而支撑学习者持续参与学习、保持学习韧性。

综上，该理论框架描绘了一个协同联动的多层次动态作用过程：人工智能系统通过多维度赋能启动了技能习得的良性循环——以精准的行为指导 KP 反馈降低学习过程中的认知负荷，进而增强学习者的掌控感与成就感，最终激发其内在动机。而被强化的内在动机，又将推动学习者投入更多练习时间，持续练习恰是技能深度习得的核心驱动力。为将上述理论框架的原则应用于实践，下面将详细阐释本研究所开发的人工智能反馈系统的核心架构与设计理念——该系统正是前述理论逻辑的具象化实现，旨在将“行为—认知—动机”的协同机制转化为可操作的技术方案。

2.基于姿态识别的实时运动反馈系统

2.1 RTMPose

为构建一个高效、精准的实时动作反馈系统，并将其应用于壮族导引术“和嘘勒”的教学实践中，本研究采用了由上海人工智能实验室与 OpenMMLab 团队共同开发的开源姿态估计框架 RTMPose。与广泛应用的 MediaPipe 相比，RTMPose 凭借其创新的 SimCC 坐标分类策略与轻量级的 CSPNeXt 主干网络，在精度与效率的权衡上实现了突破。RTMPose 在 COCO、CrowdPose 等多个权威基准数据集上取得了领先的性能，其姿态估计准确率（AP）显著优于同期的其他方案，同时能在中端硬件上实现数百 FPS 的推理速度，满足了实时系统对低延迟与高吞吐量的严苛要求。此外，该框架通过 OpenMMLab 生态提供了强大的跨平台部署能力，并支持从 2D 到 3D 的全身姿态估计，为后续的运动学分析提供了更丰富的数据基础，特别适合于需要对细微姿势偏差与动作连贯性进行量化评估的教学场景[10]。如图 1 所示，本系统所采用的 RTMPose 全身姿态模型能够精准提取 133 个关键点，从而为后续的姿势偏差定位与动作连贯性判断提供了精细化运动学分析数据基础。

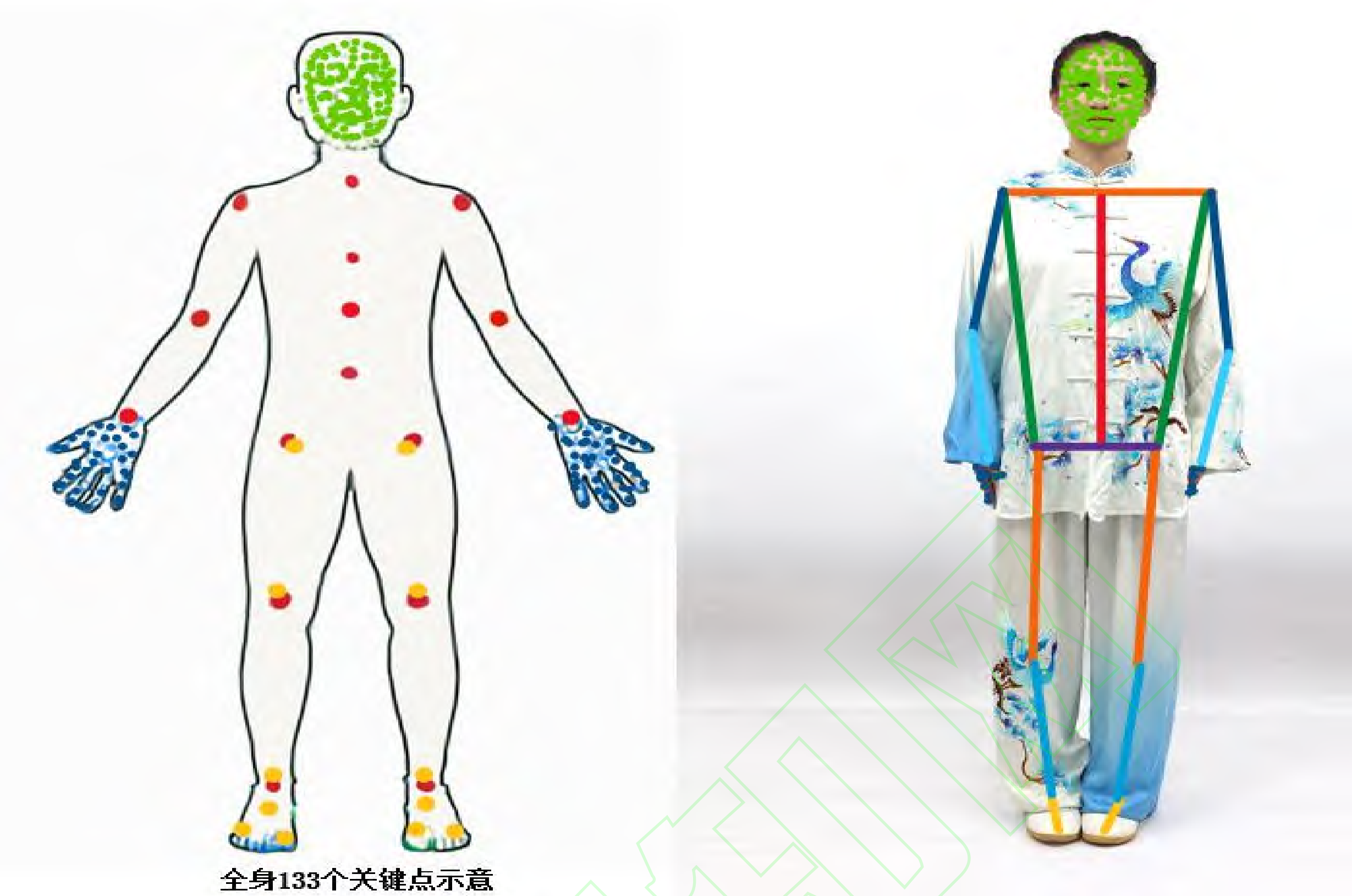

图 1 RTMPose 全身姿态模型

2.2 运动数据库的构建与验证研究

反馈系统的核心是一个动作数据库，该数据库规范了族导引・和嘘勒动作的“标准”执行方式。该动作数据库的构建与验证通过三步流程完成。该系统首先采用 RTMPose，从一个包含 28 个展示熟练族导引・和嘘勒动作练习者的视频语料库中提取原始的关键点坐标。RTMPose 所提供的全身 133 个关键点（覆盖身体、手部、面部及足部）为运动学分析提供了远超传统方案的数据维度与精度基础。随后，通过向量点积与欧几里得距离公式，从这些高精度坐标中计算出与动作质量密切相关的关键运动学特征，包括关键关节的角度及关节间的相对距离。参考已有研究方法，我们仅从丰富的关键点集合中选取每个动作中最具代表性的特征进行分析，以在保证信息全面的同时提升计算效率。图 2 展示了用于动作质量分类的关键特征。

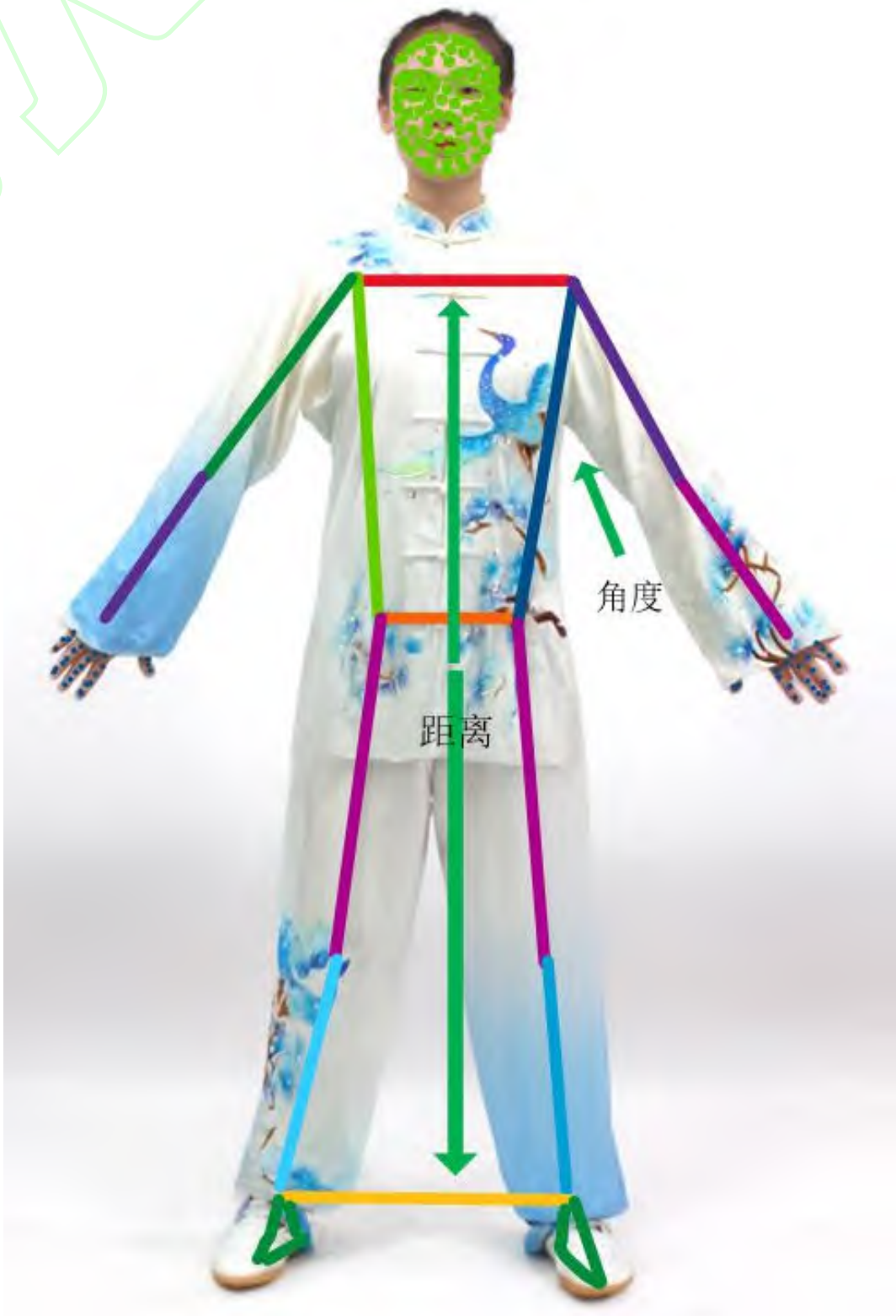

图 2 指标分析

本研究邀请国医大师等三位挖掘整理“和嘘勒”的专家，对提取出的运动学特征进行独立标注。依据运动路线及其动作幅度的要求，专家们对每个视频中练习者的动作质量进行了评估与分类，构建了一个“真实基准”数据集。由专家标注完成的数据集，作为系统自动评估动作质量的标准参考。

为验证该系统与既定标准的一致性，采用 Bland–Altman 分析方法对专家标注数据与系统标注数据之间的一致性进行了评估，结果如表 1 所示。分析显示，两种测量方法之间具有较高的一致性。所有评估动作的平均偏差均较小（范围为 –0.27° 至 7.95° ），且其 95%一致性界限在教学应用中处于可接受范围内。以导气式动作为例，其 95%一致性界限的范围为–19.46° 至 25.69° 。值得注意的是，超过 93%的差异值均落在计算所得的一致性界限之内，这一结果也印证了基于 RTMPose 的自动化测量与专家人工评估具有高度一致性，进一步验证了该系统在实际干预中的可靠性，表 1 为五种动作的 Brand–Altman 验证分析。

表 1 部分动作的 Bland - Altman 检验

“和嘘勒”动作	偏差	LOA upper	LOA lower	LOA(%) 范围
运谷式	2.86	42.64	–36.93	97.36
蓄水式	7.95	32.74	–16.92	96.03
导气式	3.15	25.69	–19.46	93.81
御龙式	4.62	28.64	–20.13	95.42
传火式	–0.27	18.29	–18.74	94.64

2.3 界面设计与功能实现

该系统采用基于网络的应用程序设计，旨在确保优异的跨平台兼容性与部署灵活性。其整体架构由两个核心模块组成：

后端：服务器端基础设施负责承载系统的核心逻辑，主要包括经过专家验证的动作数据库、用于高精度视频流处理的 RTMPose 姿态估计模块，以及最为关键的实时反馈算法。该算法基于 RTMPose 提取的 133 维全身关键点数据，通过计算关节角度、运动轨迹等精细化特征，将学习者的实时表现与数据库中的标准动作进行高精度对比与分析。

前端：客户端用户界面采用 JavaScript 开发，构建了一个支持实时学习活动的交互环境。该界面整合了网络摄像头的视频流、用于展示标准动作的教学视频播放器，以及提供即时、可视化动作反馈的显示模块。

如图 3 所示，学习者界面的设计注重直观性与引导性。左侧区域为视频播放器，用于展示由标准演练者演示的壮族导引・和嘘勒动作；右侧区域则显示学习者的实时摄像头画面，并叠加了由 RTMPose 生成的、包含身体、手部及面部细节的全身骨骼关键点模型。以实现动作姿态的可视化对比。屏幕底部列出了当前动作的关键演练标准说明。在学习者练习过程中，系统将提供即时的视觉反馈：当某项标准被成功达成并持续保持时，对应的标准描述文本将高亮为绿色。一旦当前动作的所有标准均被满足，系统将自动解锁下一个练习内容，从而构建一个结构清晰、以掌握为目标导向的渐进式学习流程。

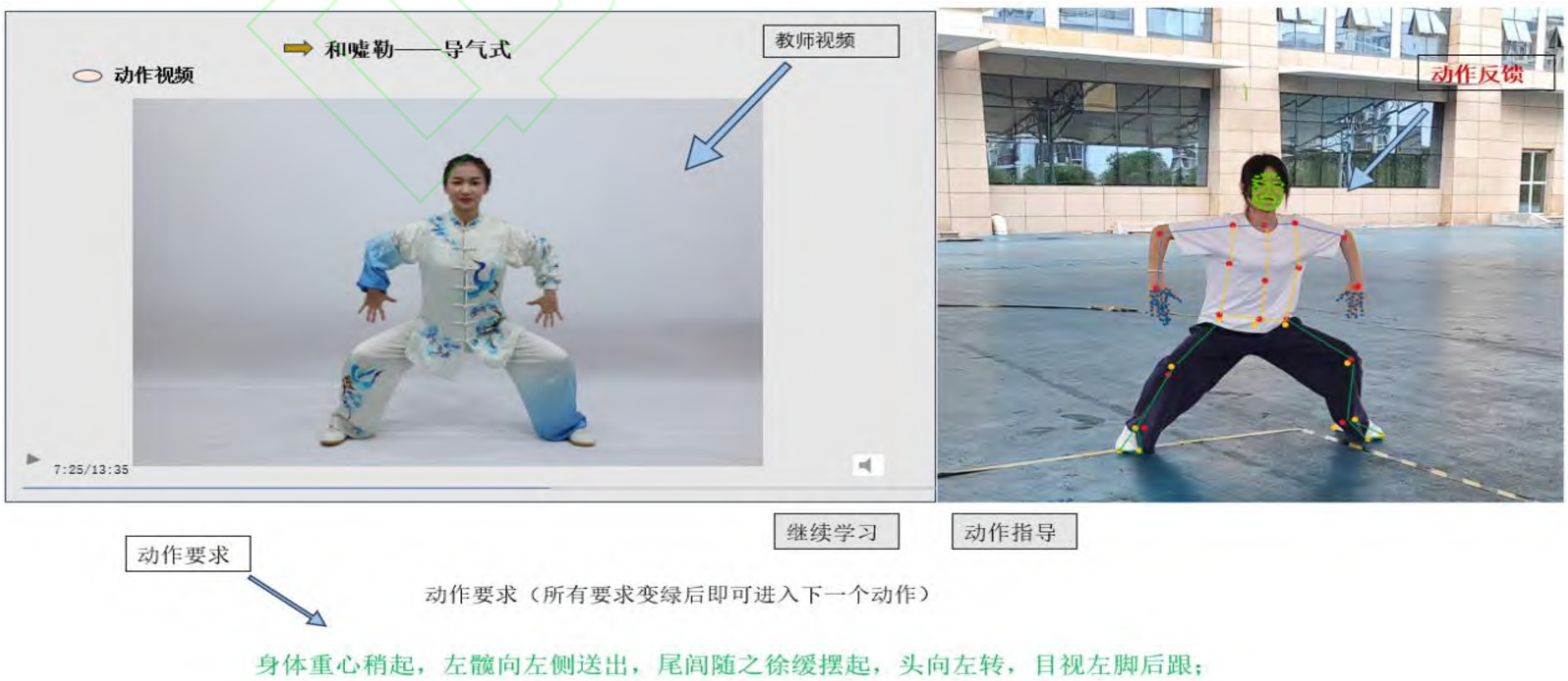

图 3 程序界面

3 研究设计与方法

3.1 研究对象

本研究以广西中医药大学大学大二公共体育传统保健体育选项课的 306 名学生为样本，进行为期 10 周的线上壮族导引・和嘘勒学习。基线评估后，将参与者分配至对照组（学习通组，

n=153）与实验组（人工智能反馈组，n=153）。

3.2 实验流程与干预方案

采用为期 10 周的前后测实验设计。第 1 周为基线期，进行统一指导与预测试。第 2–9 周为干预期，两组均需每周完成两次基于学习通平台视频的自主学习。实验组额外使用人工智能实时反馈系统辅助练习；对照组则按常规流程提交视频并接受教师延迟反馈。第 10 周进行后测，并邀请部分实验组参与者参加访谈。

3.3 测量工具

自主学习能力和学习兴趣被视为影响学习成效的核心因素[11]。因此，本研究采用三项指标评估学生的学习成果：对体育课程的兴趣、自主学习能力以及壮族导引・和嘘勒动作演练得分。此外，在线课程的学习时长被作为过程性指标加以考量。动作演练的评价标准主要包括关节角度（体现动作规格）、动作速度以及身体放松程度（反映动作演练水平）。

（1）体育学习的兴趣

学习兴趣采用《大学生体育学习兴趣量表》[12]进行评估。该量表包含五个维度，其信度与效度良好：内部一致性信度（Cronbach's α）为 0.849，结构效度经因子分析验证（KMO = 0.922）。

（2）体育教育中的自主学习能力培养

自我导向学习能力采用《大学生体育教育自我导向学习量表》[13]（吴本连，2010）进行测评。该量表包含四个维度，其信度与效度良好：内部一致性信度（Cronbach's α）为 0.891，结构效度的 KMO 指标为 0.795。

（3）壮族导引・和嘘勒演练评分标准与评估

学生的壮族导引・和嘘勒演练评分标准，包括动作规格（5 分）和动作演练水平（5 分）两个方面，满分共计 10 分。由三位国家一级武术裁判员独立对参赛视频中的套路动作进行评分，学生的最终得分为三位裁判评分的算术平均值。为确保评分者间信度，在测试前后均进行了检验，组内相关系数（ICC）分别达到 0.918（测试前）与 0.891（测试后），表明三位裁判员的评分具有高度一致性。

（4）半结构化访谈

本研究对实验组学生实施了半结构化访谈，旨在收集其对姿势识别技术辅助学习的深度反馈。访谈共包含五个开放式问题，每次访谈持续约 10 分钟。整个过程均记录参与者编号与时间戳，以确保数据的可追溯性。访谈内容由两名研究人员独立进行转录、分析与编码，所依据的是预先设定的主题框架。双方在出现编码分歧时，通过重新审阅原始记录并讨论以达成共识。

（1）请谈谈您此前参与线上体育课程的学习体验，以及在过程中遇到的主要挑战或困难。

（2）在使用本次课程中的姿势识别技术时，您是否遇到了任何技术性障碍？如有，请具体描述。

（3）与传统学习方式（如观看视频或面授）相比，您认为姿势识别辅助学习有哪些优势与不足？

（4）通过使用该技术，您是否感觉自身的学习兴趣与自主学习能力得到了提升？

（5）您是否愿意在未来继续使用此类技术进行运动技能学习？并请提出对该技术改进的具体建议，以及您认为这些改进将对线上体育教育产生何种影响。

3.4 数据处理与分析

采用 SPSS 28.0 和 Amos 28 软件进行数据分析。组间差异及教师与学生数据之间的关系通过独立样本 t 检验进行分析；组内前测与后测的差异则采用配对样本 t 检验进行评估。为探讨姿势识别技术对壮族导引・和嘘勒技能学习的影响是否通过学习兴趣、自主学习等变量起中介作用，本研究进行了平行中介分析，重点考察学习兴趣、自主学习以及学习时长在技能学习结果中的中介效应。

4 研究结果

4.1 基线等价性检验

为确认干预前两组被试的初始水平是否一致，本研究对体育学习兴趣、自主学习能力及壮族导引・和嘘勒技能演练水平各子维度进行了独立样本 t 检验。结果表明，在干预开始前，AI 反馈组（实验组）与仅学习通组（对照组）在所有观测变量上均未表现出统计学上的显著差异（所有 P> 0.05，见表 2 中 P0 列数据）。此结果证实了两组被试在基线水平上具有良好的等价性，满足后续比较分析的前提条件。

表 2 组内和组间比较

效果	组别	E0	E1	t0	P0	t1	P1	t2	P2
**1.体育学习兴趣**									
消极体育学习	E	2.44 ± 0.69	2.04 ± 0.93	0.63	0.53	1.93	0.06	1.36	0.182
	C	2.32 ± 0.56	2.32 ± 0.67			0.15	0.88		

效果	组别	E0	E1	$t0$	$P0$	$t1$	$P1$	$t2$	$P2$
积极体育学习	E	3.17 ± 0.43	4.03 ± 0.67	0.26	0.81	−6.03	<0.00	−5.47	<0.001
	C	3.13 ± 0.52	3.16 ± 0.54			−0.31	0.75		
运动技能学习	E	3.55 ± 0.46	4.02 ± 0.61	1.22	0.24	−3.65	<0.00	−3.77	<0.001
	C	3.37 ± 0.50	3.40 ± 0.61			−0.47	0.64		
课外体育活动	E	3.17 ± 0.64	3.77 ± 0.56	−1.11	0.26	−3.91	<0.001	−2.28	0.025
	C	3.38 ± 0.80	3.45 ± 0.48			−0.57	0.56		
体育关注度	E	2.97 ± 0.69	3.83 ± 0.62	−0.65	0.52	−5.04	<0.001	−3.63	<0.001
	C	3.12 ± 0.76	3.18 ± 0.73			−0.46	0.63		
**2.体育自主学习**									
学习动机	E	3.27 ± 0.54	3.93 ± 0.60	0.54	0.61	−4.13	<0.001	−4.51	<0.001
	C	3.23 ± 0.52	3.32 ± 0.43			−0.71	0.47		
学习过程	E	3.57 ± 0.53	3.81 ± 0.56	1.86	0.08	−1.76	0.07	−2.34	0.024
	C	3.37 ± 0.46	3.51 ± 0.53			−1.12	0.26		
学习效果	E	3.42 ± 0.48	3.26 ± 0.67	0.96	0.33	0.92	0.37	−0.07	0.941
	C	3.31 ± 0.46	3.25 ± 0.32			0.42	0.68		
学习环境	E	3.96 ± 0.53	4.18 ± 0.57	1.53	0.14	−1.43	0.17	−2.03	0.047
	C	3.77 ± 0.52	3.89 ± 0.56			−0.71	0.49		
**3.和嘘勒得分**									
动作规格	E	2.71 ± 0.42	3.48 ± 0.36	1.27	0.22	−8.42	<0.001	2.27	0.027
	C	2.56 ± 0.36	3.26 ± 0.36			−10.97	<0.001		
演练水平	E	2.59 ± 0.38	3.44 ± 0.38	0.83	0.42	−12.83	<0.001	2.43	0.017
	C	2.54 ± 0.36	3.22 ± 0.36			−10.66	<0.001		
总分	E	5.32 ± 0.83	6.91 ± 0.72	1.06	0.28	−12.64	<0.001	2.44	0.017
	C	5.11 ± 0.74	6.47 ± 0.71			−11.14	<0.001		
**4.参与学习时长**									
持续时间	E	–	23.85 ± 3.47	–				2.48	0.017
	C	–	21.24 ± 4.64	–					

注：E 为实验组；C 为控制组；E0 为试验前；E1 为试验后；$t0$ 为基线 t 值；$P0$ 为基线 P 值；$t1$ 为干预前后比较的 t 值；$P1$ 为干预前后比较的 P 值；$t2$ 为组间比较的 t 值；$P2$ 为组间比较的 P 值。

4.2 干预效果

4.2.1 组内变化

配对样本 t 检验结果显示，在干预后 AI 反馈组在体育学习兴趣的多数维度（除“消极体育学习”外）、学习动机以及壮族导引・和嘘勒演练水平得分上，从试验前（E0）到试验后（E1）均呈现显著提升，差异具有统计学意义（P< 0.001）。相比之下，学习通组仅在壮族导引・和嘘勒演练水平表现出显著提高（P<0 .001），而在体育学习兴趣及自主学习动机等维度未见显著变化（P> 0.05）（见表 2，$P1$ 列）。

4.2.2 组间后测比较

通过独立样本 t 检验对两组后测结果进行比较见表 2。心理指标结果：AI 反馈组在体育学习兴趣的四个维度（积极体育学习：t = −5.47，P< 0.001；运动技能学习：t = −3.77，P< 0.001；课外体育活动：t = −2.28，P = 0.025；体育关注度：t = −3.63，P< 0.001）和体育自主学习的三个维度（学习动机：t = −4.51，P< 0.001；学习过程：t = −2.34，P = 0.024；学习环境：t = −2.03，P= 0.047）上的得分均显著高于仅参加学习通对照组。在壮族导引・和嘘勒演练方面：AI 反馈组（$\bar{x}$= 6.91，S= 0.72）的总得分显著高于仅使用学习通组（$\bar{x}$= 6.47，S = 0.71），t= 2.44，P = 0.017。这种优势在动作规格（t = 2.27，P= 0.027）和演练水平（t = 2.43，P= 0.017）方面均保持一致。学习时长：行为方面，AI 反馈组（$\bar{x}$= 23.85，S= 3.47）用于练习的时间显著多于仅使用学习通组（$\bar{x}$= 21.24，S= 4.64），t= 2.48，P= 0.017。

4.2.3 中介效应分析

为探讨姿势识别技术对技能得分的影响机制，本研究构建了以自主学习能力、学习兴趣与学习时长为中介变量的多重中介模型（见图 4）。

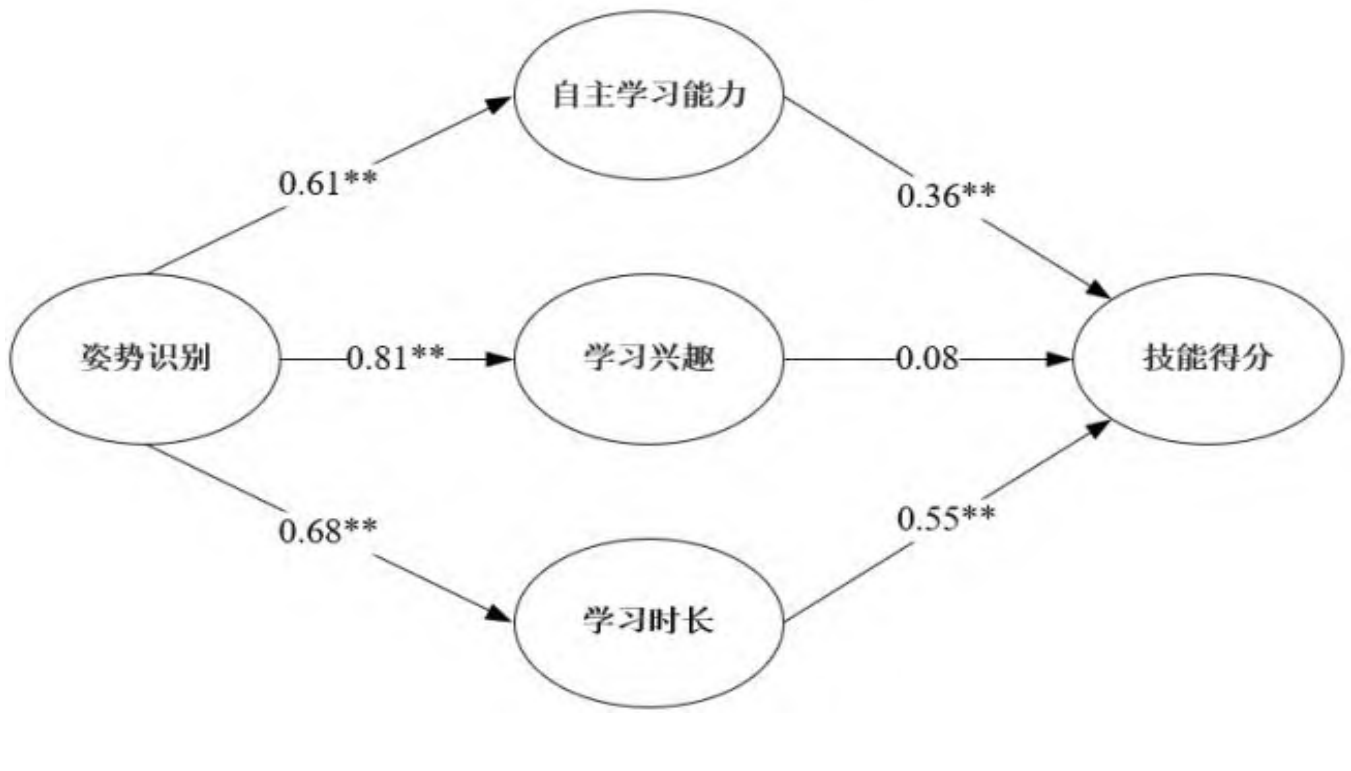

注：**P<0.001；*P<0.05。

图 4 中介效果路径

采用 Bootstrap 法进行检验（重复抽样 5000 次），结果显示（见表 3）：姿势识别对技能得分的总效应显著（b= 0.635, 95% CI [0.110, 0.887]），而其直接效应不显著（b= −0.025, 95% CI [−0.296, 0.267]），表明姿势识别对技能得分的影响完全由所设中介变量共同中介。在特定中介路径方面，学习时长（b = 0.374, 95% CI [0.267, 0.974]）与自主学习能力（b= 0.220, 95% CI [0.130, 0.727]）的间接效应均显著；而学习兴趣的间接效应不显著（b= 0.057, 95% CI [−0.154, 0.323]）。总间接效应值为 0.657（95% CI [0.129, 0.941]），进一步支持了中介路径的整体显著性。

表 3 间接效果分析

变量关系	效应值	Bootstrap SE	Bootstrap 95% CI
**间接效果**			
姿势识别→自主学习能力→技能得分	0.220	0.083	[0.130,0.727]
姿势识别→学习兴趣→技能得分	0.057	0.106	[−0.154,0.323]
姿势识别→学习时长→技能得分	0.374	0.105	[0.267,0.974]
总间接效果	0.657	0.178	[0.129,0.941]
**直接效果**	−0.025	0.141	[−0.296,0.267]
**总效果**	0.635	0.192	[0.110,0.887]
**对比差异**			
自主学习能力-学习兴趣	0.297	0.115	[0.073,0.580]
自主学习能力-学习时长	−0.015	0.126	[−0.218,0.443]
学习兴趣-学习时长	−0.293	0.108	[−0.639,−0.063]

对中介路径的对比分析发现（见表 3），“自主学习能力—学习兴趣”路径间的差异显著（b= 0.297, 95% CI [0.073, 0.580]），“学习兴趣—学习时长”路径间的差异亦显著（b= −0.293, 95% CI [−0.639, −0.063]），而“自主学习能力—学习时长”路径间的差异未达显著水平（b= −0.015, 95% CI [−0.218, 0.443]）。这表明，自主学习能力与学习兴趣、学习兴趣与学习时长在中介效应强度上存在显著差异。

综上所述，本研究验证了学习时长与自主学习能力在姿势识别技术与运动技能表现之间起完全中介作用，即人工智能反馈系统主要通过提升学生的练习投入与自主学习水平来促进其技能发展。

4.2.4 访谈结果

为深入理解人工智能实时反馈系统的教学效果，本研究对实验组参与者进行了半结构化访谈。通过对访谈文本的主题分析，我们提炼出以下四个核心主题，这些发现有效补充并解释了前述定量研究的结果。

（1）传统在线体育教育的“双刃剑”：便捷性与反馈缺失的矛盾

在探讨人工智能系统之前，学生普遍认为传统在线体育教育是一把“双刃剑”。尽管其学习便利性与时间灵活性获得广泛认可，但“缺乏实时指导与互动”成为最突出的短板。这种即时反馈的缺失导致学生在练习过程中产生不确定感，并影响学习效率。例如，有学生表示：“练习时我常常无法判断动作是否准确，没有即时反馈，学习效率很低”（N02）。另一位学生进一步指出：“最大的困难在于无法自我判断动作正误，很多错误难以自我察觉”（N21）。此外，互动不足也带来了情感上的孤立感，如有受访者提到：“很怀念以前和同学一起训练的互动氛围，现在感觉特别孤单”（N22）。

（2）人工智能作为“私人教练”：实时反馈的价值与技术局限

多数学生将姿势识别系统视为弥补传统在线学习不足的关键工具，并将其比作“私人教练”，认为其实时纠错功能具有重要价值。一名受访者表示：“姿势识别系统帮助我更深入地理解动作，并能实时修正错误”，并强调“这对壮族导引・和嘘勒这类强调动作精准性的内容尤为重要”（N01）。系统的实时评估功能也被视为

技能提升的关键机制，如有参与者指出：“技术让我发现了很多之前没意识到的动作问题，有效提升了我的技术水平”（N05）。然而，学生也敏锐地指出了系统当前的技术局限，尤其是在“空间受限或执行复杂动作时识别准确率不足”的问题（N01）。除技术因素外，学生还提到系统在人性化方面的欠缺，例如“缺乏情感支持”（N19），以及在复杂情境下“无法提供直接解释或有效帮助”（N23），反映出其在教学交互与支持能力方面的不足。

（3）从被动观看到主动参与：人工智能反馈的激励作用

从被动观看视频到参与实时反馈的转变，显著提升了学生的学习动机与参与度。定量研究中观察到的自主学习能力提升，在此得到质性数据的支持。实时反馈被普遍视为重要的激励因素，有学生表示：“实时反馈增强了我的学习兴趣。系统指出错误时，我更愿意主动改正”，并认为“这项技术让我在学习中变得更主动”（N17）。这种互动机制使练习过程更具吸引力和游戏化特征，反馈信息为学生提供了清晰的进步轨迹与成就感。如一名受访者所言：“看到自己不断进步，带给我很强的成就感”（N23），这种感觉进而“促使我在课外也愿意主动练习”（N04）。

（4）未来展望：构建更智能与个性化的学习系统

学生对技术的未来发展普遍持积极态度，并提出了若干改进建议。核心议题包括提升系统识别精度与智能化水平，如有学生希望“系统在识别细微和快速动作时更加精准”（N03）。此外，部分学生期待系统具备更强的互动性与适应性，建议“引入虚拟教练功能”（N06），或开发能“根据个体体能和习惯进行个性化适配”的模式（N14）。总体来看，学生认为随着技术完善，该系统有望通过提供个性化实时反馈，显著推动在线体育教育发展，其应用前景可拓展至武术、常规健身等多种运动场景。

5 讨论

5.1 主要研究发现概述

本研究通过为期 10 周的教学实验证实，在线上壮族导引・和嘘勒课程中，整合人工智能实时反馈系统能显著提升学生的运动技能表现，其效果优于仅使用学习通平台的传统模式。中介效应分析进一步揭示了其内在机制：系统主要通过增强学生的自主学习能力并延长其有效学习时长来促进技能掌握，而学习兴趣的提升并未表现出显著的直接中介作用。这一量化结论在后续的质性访谈中得到了充分印证，学生们普遍认为 AI 反馈在激发练习动力和提供精准指导方面具有核心价值。

5.2 人工智能反馈在运动技能学习中的催化作用

本研究核心发现在于，AI 驱动的实时反馈系统能有效弥补传统在线体育教学中“指导缺失”的瓶颈。该结果与既有研究中关于即时反馈促进运动技能习得的结论一致[14]。并通过学生的质性反馈获得了机制性解释。学生指出，传统模式下他们“难以判断自身动作是否正确”（N02），而 AI 系统通过可视化的纠错指引，将抽象的动作标准转化为可操作的具体信息，有效降低了学习过程中的不确定性。这一过程深刻契合运动技能学习理论。系统提供的即时、具体的外部反馈，加速了学习者动觉意识（本体感觉）的形成与内部参照的建立，正如学生所言“意识到许多以往未曾察觉的错误”（N05）。然而，学生们也客观指出了当前技术的局限，如复杂场景下的识别误差及“缺乏情感支持”（N19）。这表明，AI 系统在担任“技术教练”方面表现出色，但在情感激励与复杂问题阐释方面尚无法替代人类教师。因此，其最佳定位应是作为传统教学的有力补充，为实现规模化基础上的个性化反馈提供可行路径。

5.3 行为的中介作用：实践优先性的解析

本研究通过中介分析，揭示了一条“认知减负—动机激发—行为投入”的因果路径。AI 干预同时提升了学生的学习兴趣，但仅有自主学习能力与学习时长是技能提升的显著中介变量。这表明，在技能形成的初期阶段，有效练习的积累与自主学习能力的发展是技能提升的关键。

本研究构建的理论链条如下：首先，AI 反馈通过提供精准的动作表现反馈，显著降低了学生在自我评估时产生的额外认知负荷，这与认知负荷理论的核心观点高度契合。认知负担的减轻使练习过程更为流畅，为持续参与创造了前提。其次，这种流畅的练习体验以及进步的可视化（如“看到自己不断进步，带给我很强的成就感”N23），有效满足了学生的能力感需求，从而激发了其内在动机。最终，这种动机并非直接转化为技能，而是转化为更强的学习自主性与更长的练习时间。因此，AI 系统的成功，关键在于它将激发出的心理动力，高效地转化为了实质性的行为投入与自我调控能力。

5.4 对理论与实践的启示

理论启示方面，本研究构建并验证了一个整合认知与动机理论的序列中介模型，清晰地刻画了 AI 反馈促进技能学习的内在路径。同时，它对自我决定理论在新手技能学习情境下的应用提供了重要补充：在此阶段，能力需求的满足可能主要通过促进自主学习能力与持续的行为投入来间接影响学习成果。

实践启示方面，为在线体育教育平台的设计提供了明确方向：（1）功能设计应超越表面的“游戏化”，聚焦于通过精准反馈切实降低学生的认知负荷，核心目标是提升学生的自主学习能力并增加有效练习时间。（2）教学模式应采用人机协同的混合式教学。AI 负责高频、精确的重复训练反馈，而人类教师则专注于情感激励、策略指导与人文关怀，实现优势互补。

5.5 研究局限与未来展望

本研究存在若干局限，未来研究中需要加以突破：（1）样本与生态效度：样本仅来自一所大学，且学习内容为结构化的传统保健体育。未来研究需检验该模式在 K-12 学生、老年群体或开放式运动技能（如球类）中的普适性。（2）长期效果未知：10 周的干预周期足以观测短期习得，但无法评估技能的长期保持与迁移效应。需开展纵向追踪研究，并排除“新奇效应”的潜在干扰。（3）统计检验力：尽管中介分析揭示了自主性学习和学习时长的关键作用，但较小的样本量可能限制了检测其他心理变量（如学习兴趣）微弱中介效应的统计检验力。未来需要在更大样本中验证本研究的理论模型。（4）技术迭代：学生反馈揭示了系统在识别准确性与交互智能方面的不足。未来的技术优化应集中于提升复杂动作的识别鲁棒性，并探索集成自适应学习与情感计算的新一代智能辅导系统。

6 结论

本研究证实，人工智能实时反馈系统能够通过“认知减负-动机激发-行为投入”的作用链条，有效提升在线运动技能学习的质量与效果。其核心价值不仅在于营造更具吸引力的学习环境，更在于能将学生的参与动机转化为持续的自主学习行为与有效的练习投入——这是技能精熟的根本保障。展望未来，AI技术与人类智慧深度融合的混合教学模式，构成了在线体育教育发展的前瞻性范式：技术系统承担起可规模化的精准反馈职责，而教师则专注于启迪、激励与关怀，共同构建一个既高效又充满人文温度的下一代体育学习生态系统。

参考文献

[1]Lin Y N, Hsia L H, Hwang G J. Fostering motor skills in physical education: A mobile technology-supported ICRA flipped learning model[J]. Computers & Education,2022:177.
[2]Huang A, Lu O, Yang S. Effects of artificial Intelligence-Enabled personalized recommendations on learners? learning engagement, motivation, and outcomes in a flipped classroom[J].Computers & Education,2023: 194.
[3]韦东谊,陈立群,韦健,等.“和嘘勒—导引术”壮医养生价值探析[J].体育科技,2024,45(1): 21-23.
[4]Lambert K, Hudson C, Luguetti C. What would bell hooks think of the remote teaching and learning in Physical Education during the COVID-19 pandemic? A critical review of the literature[J].Sport Education and Society,2024,29(6): 667-683.
[5]Baker S, Anderson J, Burke R, et al. Equitable teaching for cultural and linguistic diversity: exploring the possibilities for engaged pedagogy in post-COVID-19 higher education[J]. Education Review, 2022,74(3): 444-459.
[6]Hsia L H, Hwang G J, Hwang J P. AI-facilitated reflective practice in physical education: an auto-assessment and feedback approach[J]. Interactive Learning Environments,2024, 32(9): 5267-5286.
[7]Wang Y P, Wang X Y. Artificial intelligence in physical education: comprehensive review and future teacher training strategies[J]. Frontiers in Public Hetalth,2024:12.
[8]Zhu B W, Kaber D, Zahabi M, et al. Effects of feedback type and modality on motor skill learning and retention[J]. Behaviour & Information Technology,2020,39(4):431-442.
[9]Ryan R M, Deci E L. Self-determination theory and the facilitation of intrinsic motivation, social development, and well-being[J]. American Psychologist,2000,55(1):68-78.
[10]Saurav S, Gidde P, Singh S. Exploration of deep learning architectures for real-time yoga pose recognition[J]. Multimedia Tools and Application,2024.
[11]Ardito G, Czerkawski B. The Development of Autonomous Student Learning Networks: Patterns of Interactions in an Open World Learning Environment for Teachers Exploring Teaching with and through Computer Science[J]. Sustainbility,2021,13(16).
[12]顾海勇,解超.大学生体育学习兴趣评价量表的编制[J].广州体育学院学报. 2012,32(3): 122-124.
[13]吴本连.自主学习方式影响大学生体育学习效果的实验研究[D].华东师范大学,2010.
[14]Hsia L H, Hwang G J. From reflective thinking to learning engagement awareness: A reflective thinking promoting approach to improve students' dance performance, self-efficacy and task load in flipped learning[J].British Journal of Educational Technology,2020,51(6):2461-2477.
