# Supplementary material for: Effects of AI-mediated real-time feedback on motivation and motor skill learning in traditional ethnic sports education: testing self-determination theory mechanisms
Source: Front Psychol. 2026 Oct 16;17:1958348. doi: 10.3389/fpsyg.2026.1958348 (PMC13720675; doi:10.3389/fpsyg.2026.1958348)
Supplement: Supplementary file 4 [file Data_Sheet_4.PDF]

DOI：10.14038/j.cnki.tykj.2026.03.035　网络首发时间：2026-04-30 09:57:09　网络首发地址：https://link.cnki.net/urlid/45.1171.G8.20260429.1633.070

责任赋予对大学生传统保健体育课程满意度的影响路径研究

韦东谊[1]　杨　宇[2]　陈桂真[1]　覃月泉[1]　李荣源[1]

（1.广西中医药大学 体育部， 广西 南宁 530200；2.广西中医药大学 第一附属医院， 广西 南宁 530001）

摘要：基于自我决定理论，文章构建了课程评价中责任赋予对传统保健体育课程满意度的结构方程模型，旨在检验其影响机制。作者采用问卷调查统计法对 5 所高等中医药院校大一 343 名学生的传统保健体育课责任感知量表、基本心理需求满足量表、动机水平量表以及传统保健体育课满意度量表进行测量和分析。结果显示，模型与观测数据具有良好的拟合度。研究发现，在课程评价过程中赋予学生更多责任，有助于满足其基本心理需求，从而促进自主性动机的发展。研究证实了赋予大学生责任通过胜任感、自主性与关联性需求的满足激发内在动机显著提升大学生对传统保健体育课的整体满意度，为体育课程评价体系改革提供理论依据。

关键词：自我决定理论；责任赋予；基本心理需求；自主动机；课程满意度；传统保健体育

传统保健体育源远流长，其理论基础根植于中医学，以导引、武术为核心锻炼方法，旨在强身健体、修身养性、延年益寿。作为高等中医药院校的特色课程，传统保健体育兼具显著的健身价值与深厚的文化传承意义。其教学核心在于通过“课内触发–课外延伸”机制，有效促进学生运动习惯的可持续养成[1]。体育教师是传统保健体育活动开展的主要推动者之一，因此，教师的教学举措对于学生能否提高其日常体育活动水平至关重要。还有研究表明，教师采用具有积极心理影响的策略，比如在体育课上提高学生的内在动机，将有助于培养和强化与体育活动相关的行为习惯。

动机作为多维度交互作用的动力系统，通过生理激活、情感驱动、认知评估与社会交互的协同作用，动态调节行为表现。具体表征为行为持续周期的延展性、执行强度的梯度性以及发生频次的节律性。这些构成要素间形成的非线性交互作用，不仅递归式调节行为动力学的矢量方向，更通过自我强化循环机制产生涌现性特征，最终实现行为的正向增益、稳态维持或负向衰减。

本研究聚焦于自我决定理论[2]，在自我决定理论中，通过连续体模型阐释行为自我决定性的差异层次，将动机类型划分为三级谱系：（a）无动机状态（行为参与缺乏内在驱动力）；（b）受控动机（受外部奖惩机制支配的外源性驱动）；（c）自主动机（源于内在兴趣的内生性驱动）。在自我决定理论中确立三个核心的基本心理需求维度：自主性需求（渴望自主参与活动的意愿）、胜任感需求（追求有效互动的能力感知）以及关联性需求（寻求群体归属的情感诉求）。这些心理变量通过需求满足度来介导不同动机类型的转化路径。现有研究表明，基本心理需求作为中介变量，可显著正向预测自决性动机的形成概率。

由此提出动机层级模型[3]，根据该模型动机可划分为三个层级：整体动机、情境动机与具体场景动机，三者通过层级嵌套机制产生交互影响。该模型揭示环境社会因素作为背景变量，其作用效力取决于自主性–胜任感–关联性三维需求的满足阈值[4]。当需求满足度达到临界值时，将触发内在动机的强化机制，进而在认知重构（如运动技能内化）、情感体验（如愉悦感提升）及行为模式（如运动频率增加）三个层面产生显著的正向溢出效应[5]。

在教育实践中，责任赋予型教学氛围被证实能有效激活学生的运动认知图式[6]。不同研究表明，当教师给予学生自主权和责任时，学生对体育课的重视程度会更高，他们从中获得的乐趣也会增加。在一项以 532 名学生开展的研究中，采用任务自主性差异范式发现：在教师实施结构性赋权的情境下，表现出典型的自决动机特征，其需求满足度提升，学生体育课程参与度增加，课外自主运动频率提升[7]。

教学评价可被视为一种用于监测和衡量学生学习效果的重要手段。教师可以采用一种更具控制性的教学方式，即更注重结果而非学习过程，或者采用一种更有利于学生自主性的教学风格，让学生参与到自己的学习过程中，诸如自我评估、共同评估或他人评估等方法。考虑到学习过程对学生取得成果的重要性。在本研究中，采用了赋予学生责任感知作为动机变量的前置变量进行评价。

万来红等人的研究指出[8]，学生们乐于通过不同的策略和选择来实现他们的目标，这反过来又激发了课堂中的内在动机，从而增加了学生在

基金项目：广西壮族自治区高等教育本科教学改革工程项目“‘壮族导引术’融入高校公共体育课程的研究与实践”（编号：2025JGB258）。

第一作者简介：韦东谊（1973—），学士，副教授，研究方向为传统保健体育、运动训练与教学。

课堂外锻炼的可能性。研究表明，学生参与评估提高了他们对学习的投入，以及学生参与不同评估策略的重要性[9]，且自主性支持增加，课堂愉悦指数增长[6]。还有研究强调体育教育与运动应以学生自主以及明确学生责任为导向的重要性，还指出，一种能让学生在课堂上有机会选择、参与并做决策的教学方式，会带来更愉快的参与体验，并增强学生的内在动力。因此，通过赋予责任让学生获得参与的机会。

基于动机层级的理论框架，本研究将传统保健体育课程满意度设定为因变量。根据葛耀君等人[10]的观点，从一项活动中获得的愉悦感，即与快乐和幸福感，能让人长期保持参与度，提高依从性，并使其成为生活方式不可或缺的一部分。同样，愉悦感可被理解为学生对体育课上所开展活动的重视感，并且对体育课的满意度与取得更好的学业成绩相关[5]。有研究显示，为避免学生放弃体育活动，教师必须努力让教学活动变得有趣，避免无趣的活动[11]，因此教师开展教学活动中起着至关重要的作用。胡小清等人研究认为，当学生获得乐趣时，他们往往会产生内在动力，并更加重视这门课程。对体育课的满意度将与基本心理需要的满足以及更自主的动机呈正相关[5]。

近年研究揭示体育教育中动机支持策略的重要作用中，Van Doren 等人[12]研究认为，满足学生基本心理需求的课程设计，可显著提升学习效能、运动愉悦感及行为持续性，其效应量在教育干预研究中达到中等以上水平。此外，张宝根等人[13]研究发现最具自主性的动机形式能正向预测学生对体育课的重视程度，进而也能正向预测学生对体育课的满意度。

关于体育课程的满意度、参与积极性与厌倦感，有研究表明高度的自我决定感与更高的学习努力程度、更强的愉悦体验、对体育课程重要性的认同和积极行为的形成密切相关[9]。相反，若缺乏自我决定性的内在动机，其结果往往表现为负面情绪，例如在课堂中产生厌倦感。

因此，本研究旨在通过构建结构方程模型，探讨在传统保健体育评价过程中赋予学生责任对基本心理需求、自主动机以及大学生对课程满意度之间关系的影响机制，据此提出以下具体假设：

假设 H_1：传统保健体育课程评价中赋予学生责任对基本心理需求中的自主性、胜任感和关联性产生正向的预测效应；

假设 H_2：基本心理需求的自主性、胜任感和关联性满意度将对自主动机产生正向预测效应；

假设 H_3：自主动机将对学生在传统保健体育课的满意度产生正向预测效应。

假设 H_4：基本心理需求（自主性需求、胜任感需求和关联性需求）在传统保健体育课程评价中赋予学生责任与传统保健体育课的满意度之间存在中介效应。

假设 H_5：基本心理需求（自主性需求、胜任感需求和关联性需求）、自主动机在传统保健体育课程评价中赋予学生责任与传统保健体育课的满意度之间存在链式中介效应。

1 研究对象与方法

1.1 研究对象

本研究选取上海中医药大学、广西中医药大学、黑龙江中医药大学、河南中医药大学、贵阳中医药大学，5 所中医药院校大一学生各 80 名，共发放问卷 400 份，收回有效问卷 343 份，回收率 85.75%，其中男生 144 名，女生 199 名。

1.2 研究工具

1.2.1 赋予学生责任量表

赋予学生责任量表，借鉴 Hsu 等人编制的《体育课学生责任量表》修改而成，用来测量大学生在传统保健体育课程评价中对自身所承担责任水平的认知（例如与体育老师共同确定我的分数很重要）。该因子由 5 个题目构成，验证性因素分析（CFA）结果显示适配度较好：χ^2=28.84，df=5，P<0.001，χ^2/df=5.77，CFI=0.93，GFI=0.95，AGFI=0.91，RMSEA=0.07。

1.2.2 基本心理需求量表

基本心理需求量表，借鉴王景贤（2019）编制[14]的《青少年体育健身运动基本心理需求量表》改编而成，用来测试大学生基本心理需求的满足程度。该量表由 12 个题目组成，分为 3 个维度。每个维度由 4 个题目构成，其中自主性需求，（例如，我所进行的太极架势符合我的运动能力）；胜任感需求，（例如，我能有效地完成太极拳）关联性需求（例如，我觉得能够与同伴坦诚交流）。验证性因素分析（CFA），结果显示出较好的适配指数：

χ^2=277.92，df=51，P<0.001，χ^2/df=4.47，CFI=0.93，GFI=0.92，AGFI=0.93，RMSEA=0.06。

1.2.3 动机水平测量表

动机水平测量表，本研究聚焦于自我决定动机水平，借鉴 Goudas 编制[15]《体育课中感知因果源量表》的修改版，用于衡量大学生自我决定动机水平，该量表由内在动机（例如，因为我喜欢学习太极拳）和认同调节（例如，因为在传统保健体育课上表现出色对我很重要）组合而成。通过验证性因素分析（CFA），结果显示出较好的适配指数：χ^2=65.96，df=20，P<0.001，χ^2/df=3.298，CFI=0.97，GFI=0.95，AGFI=0.92，RMSEA=0.06。

1.2.4 传统保健体育课的满意度量表

传统保健体育课的满意度量表，借鉴史青（2010）编制[16]的“大学生体育课学习满意度量表”修改版，用于衡量学生在传统保健体育课上

表现出的满意度，该因子包含 5 个题目（例如，通常我觉得传统保健体育课很有趣）。在验证性因素分析（CFA）中，结果显示出良好的适配指数：χ^2=2.718，df=5，P<0.743，χ^2/df=3.36， CFI=0.99，GFI=0.99 ，AGFI=0.99，RMSEA=0.001。

在以上所有问卷中，学生针对所有题目的回答均采用 5 点李克特量表范围从 1 到 5，1 表示学生完全不同意，5 表示学生完全同意。

1.3 数据统计与分析

采用 SPSS 25.0 和 AMOS 28.0 软件进行数据处理与统计分析。首先，采用可靠性分析和验证性因子分析对各变量量表进行信度和效度检验。其次，采用探索性因子分析对调查问卷所有测量题项进行共同方法偏差检验；随后，通过均值、标准差以及皮尔逊相关分析对各变量之间的相关关系进行统计。最后，采用 AMOS 28.0 软件构建结构方程模型，运用 Bootstrap 检验分析等统计方法进行中介效应检验，抽样次数设置为 5000，置信区间为 95%。

2 研究结果

2.1 共同方法偏差检验

在运用问卷调研法进行数据采集的过程中，可能面临共同方法偏差的挑战。为缓解该偏差对研究结果的影响，本研究参考 Podsakoff 提出的方法，数据分析显示，Harman 单因素检验提取的 6 个因子特征值均大于 1，且首个因子的解释方差（39.18%）未超过 40%的临界值。据此，本研究不存在显著的共同方法偏差问题。

2.2 测量模型评估

如表 1 所示，本研究量表是 6 个维度共计 30 项题目，各题目因子载荷系数分布于 0.61～0.90（P<0.05），有统计学意义。组成信度（CR）大于 0.7。各维度收敛效度（AVE）分布在 0.47～0.67 大部分大于 0.5 以上，0.47 在可接受的范围。根据 Fornell &Larcker（1981）的建议在对角线 AVE 开根号值大于其它构面的相关，因此代表区别效度存在。

表 1 研究构面信度，收敛与区别效度分析表

维度	题目	因素负荷量	组合信度 CR	收敛效度 AVE	区别效度 1	2	3	4	5	6
1.责任赋予	5	0.61–0.75	0.82	0.47	**0.69**					
2.课堂满意	5	0.69–0.90	0.91	0.67	0.25	**0.82**				
3.关联性	4	0.63–0.88	0.84	0.58	0.28	0.56	**0.76**			
4.胜任感	4	0.62–0.84	0.82	0.54	0.36	0.68	0.66	**0.73**		
5.自主需求	4	0.64–0.84	0.81	0.52	0.36	0.70	0.52	0.63	**0.72**	
6.自主动机	8	0.62–0.83	0.91	0.55	0.31	0.64	0.52	0.65	0.71	**0.74**

注：CR 为组成信度（Composite Reliability）；AVE 为平均方差萃取量(Average Variance Extracted)；对角线粗体字为 AVE 开根号值，下三角为维度的皮尔森相关。

2.3 描述性和可靠性统计

将传统保健体育课程评价中赋予学生责任，基本心理需求的自主性需求、胜任感需求、相关性需求，自主动机，以及课堂满意度分析，结果表明各变量之间呈显著正相关关系，且 P＜0. 01（如表 2）。

表 2 各变量描述性统计与相关分析表

变量	$\bar{x}$	SD	1	2	3	4	5	6
1.自主动机	3.59	0.91						
2.自主性需求	2.85	0.88	0.60**					
3.胜任感需求	3.55	0.98	0.63**	0.52**				
4.相关性需求	3.81	0.92	0.51**	0.46**	0.56**			
5.赋予学生责任	3.47	0.95	0.32**	0.24**	0.34**	0.25**		
6.体育课堂满意	3.73	1.05	0.71**	0.63**	0.65**	0.53**	0.23**	

注：**代表 P<0.01。

2.4 结构方程分析

本研究以传统保健体育课程评价中赋予学生责任的认知为自变量，传统保健体育课满意度为因变量，基本心理需求（自主性需要、胜任感需求和关联性需求）和自主动机为中介变量，构建结构方程模型。采用 Amos 28.0 进行数据分析，结果显示，χ^2=1055.615，df=395，χ^2/df=2.672，P<0.001，RMSEA=0.056，CFI=0.921，GFI=0.912，AGFI=0.931，模型拟合良好[17]，如图 1 所示。

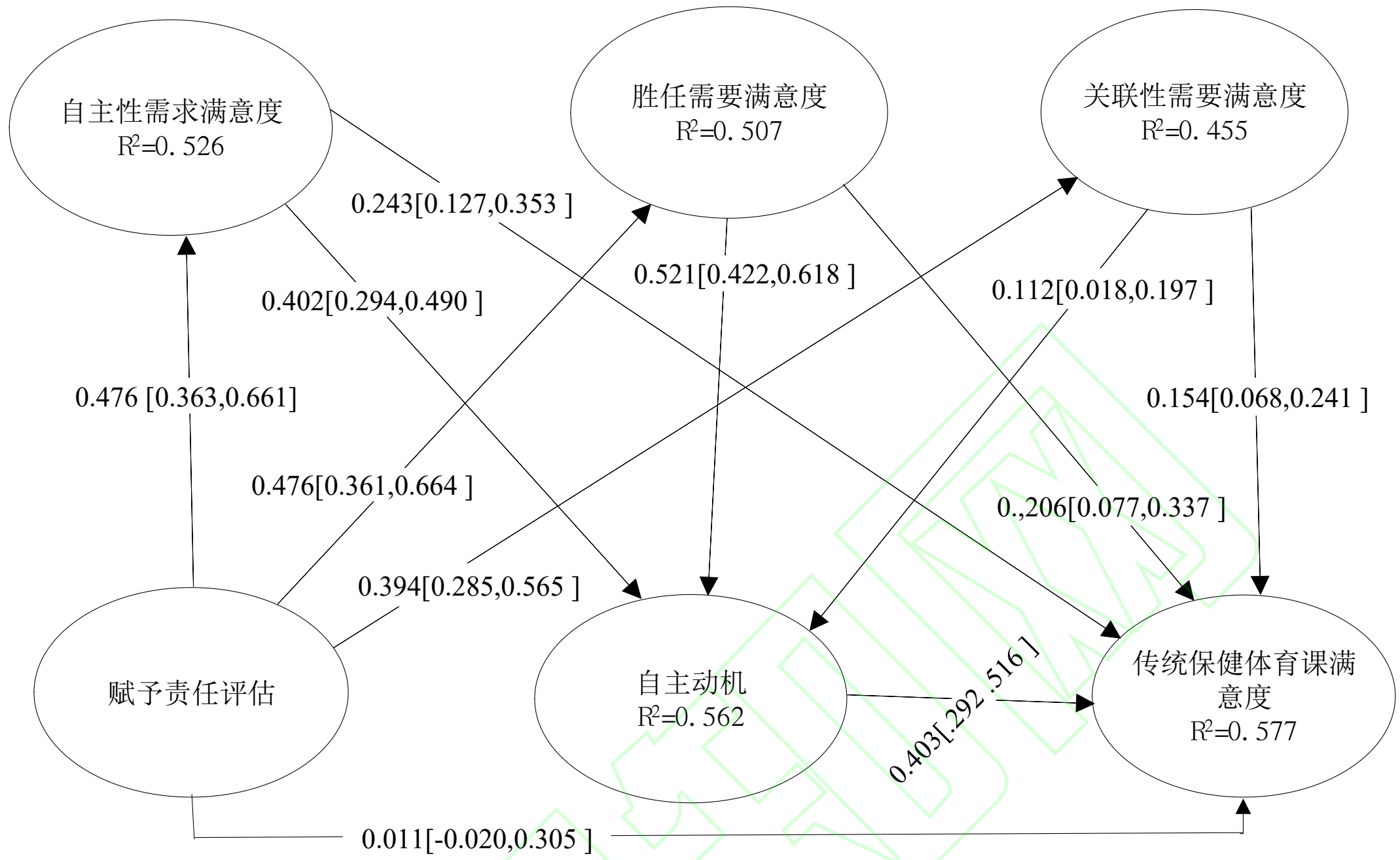

图1. 结构方程模型（SEM）:赋予大学生责任→基本心理需求满足→自主动机→传统保健体育课程满意度。数据为标准化参数（β[95%CI]和R²(SMC, Square Multiple Correlation）。

采用 Bootstrap 方法,通过 5000 次反复采样,并设置 95%的置信区间（不包含 0 则显著）,进行路径效应检验，通过标准化回归权重分析，检验各个因素对其他变量预测的作用。结果显示，课程评价中赋予学生责任感知正向预测自主性需求满意度（β=0.476[0.363，0.661]）、胜任感需求满意度（β= 0.476[0.361，0.664]）以及关联性需求的满意度（β= 0.394[0.285，0.565]）。另一方面，自主需求满意度（β=0.402[0.294，0.490]）、胜任感需求满意度（β=0.206[0.077，0.337]）以及关联性需求满意度（β=0.1120.018，0.197]）正向预测自主动机，同时，自主动机对传统保健体育课满意度有正向且显著的预测作用（β=0.403[0.292，0.516]）。赋予学生责任对传统保健体育课堂满意度的直接效果不显著为(β=0.011[−0.020，0.305])，因此该模型是完全中介。

通过 Amos 语法定义特定间接效果，通过 Bootstrap 法（5000 次抽样）估计特定间接效应的偏差校正置信区间，进行中介效应检验，非标准化结果显示,赋予学生责任对传统保健体育课堂满意度的总间接效果为（b=0.543[0.461，0.911]）。基本心理需求在赋予学生责任评价与传统保健体育课程满意度存在中介效应，分别是经自主性需求的效应值为 0.127 占总间接效应值的 23.39%，经胜任感需求的效应值为 0.107 占总间接效应值的 19.70%，经关联性需求的效应值为 0.066 占总间接效应值的 12.12%。基本心理需求和自主动机在赋予学生责任与传统保健体育课堂满意度之间存在链式中介效应，分别是经过自主性需求和自主动机的效应值为 0.084 占总间接效应值的 15.47%;经过胜任感需求和自主动机的效应值为 0.110 占总间接效应值的 20.26%；经过自主性需求和自主动机的效应值为 0.049 占总间接效应值的 10.82%，如表 3 所示。

表 3 结构方程模型中的中介效应（非标准化）

变量	效应(b)	SE	P 值	95%CI(置信区间)	效应量（%）
**赋予学生责任–>课堂满意度**	**0.543**	**0.156**	**<0.001**	**0.461,0.911**	**100**
经自主性需求	0.127	0.048	<0.001	0.064 ,0.225	23.39

续表 3

变量	效应(b)	SE	*P* 值	95%CI(置信区间)	效应量（%）
经胜任感需求	0.107	0.052	0.004	0.041, 0.211	19.70
经关联性需求	0.066	0.031	<0.001	0.029, 0.136	12.12
经自主性需要和自主动机	0.084	0.028	<0.001	0.050 ,0.147	15.47
经胜任感需求和自主动机	0.110	0.050	<0.001	0.064 ,0.209	20.26
经关联性需要和自主动机	0.049	0.010	<0.001	0.010,0.152	10.82

3 讨论和结论

3.1 赋予责任与基本心理需求满意度之间的关系

本研究结果显示，传统保健体育评价中对赋予学生责任的感知，与基本心理需求（自主性、胜任感和关联性）的满意度之间呈正相关且具有正向显著的预测关系，与研究假设 H_1 相符。已有研究表明，当学生拥有责任选择的机会时，其技能水平、身体活动参与度以及能力感知均有所提升。还有研究表明，当学生有机会在空间、时间、器材或分组等多种方法学方面做出决策时，他们的学习参与度会更高。有研究指出，责任感对积极的心理中介变量有正向预测作用，提高其自主需求的满足感，随之提升其对体育活动的满意度和愉悦感，教师赋予责任的行为有助于学生自主性的发展，与本研究基本一致。动机包含一系列情感、认知和社会现象。研究表明，若采用一种鼓励学生积极参与教学过程的教学方式，将显著提升其在认知与身体层面的投入水平，这可以解释为何学生在参与评价过程后，表现出更高的体育课程满意度和更强的学习投入感，因为其内在动机得到了有效激发[18]。

3.2 基本心理需求满意度、自主动机与传统保健体育课的满意度之间的关系

研究结果表明，基本心理需求中的自主性、胜任感和关联性的满足对自主动机产生积极且显著的预测作用。假设 H_2 得到了支持。有研究不仅证实了责任感对心理中介因素具有正向预测作用，还进一步指出，责任感通过提升内在动机，进而正向预测学生对体育教育的重视程度，最终增强其持续参与体育活动的意愿。已有研究表明，提升学生积极性的关键在于赋予其更多责任，并采用有助于增强学生自主性的教学策略。另有研究表明，在体育技能教学中赋予学生更多的自主权时，其自主动机水平将显著提升。评价过程不应仅限于教师对成绩的评定，而应为学生提供参与和决策的机会，并考虑在教学中引入初始协议与双向沟通机制。还有研究表明，基本心理需求的满足与自主动机之间存在密切联系，这与本研究的发现一致，即基本心理需求的充分满足有助于增强个体的内在动机[5]。

本研究分析结果显示，自主动机正向且显著地预测学生对传统保健体育课的满意度。假设 H_3 得到支持。已有研究表明[13]，基本心理需求的满足能够预测个体在学习过程中表现出的较高水平的内在动机，而这通常与课堂中的积极情绪体验，如享受感和满意度的提升密切相关。因此，内在动机与课堂满意度之间存在显著关联。已有研究还进一步验证了基本心理需求对于在体育课上产生积极效果的重要性[5]。

3.3 基本心理需求在赋责任与课程的满意度之间存在中介效应

本研究结果显示，课程评价中赋予学生责任对传统保健体育满意度的影响不显著，表明直接效果不存在，说明该模型变量之间的关系是完全中介模型。研究结果还显示基本心理需求（自主性、胜任感、关联性）的满足在赋予学生责任对传统保健体育满意度之间担负中介作用，假设 H_4 得到验证，效应值 0.301，占总效应值的 55.43%。与我们的研究结果类似，Curran & Standage 研究[19]证实，学生在体育课程中对自主性、胜任感和关联性的基本心理需求的满足程度与其在体育课中的投入程度之间存在显著的正向关系。然而，本研究发现，自主性需求对体育课满意度的预测力最强。同样，研究还发现，青少年在体育课上对自主性、胜任感和关联性的基本心理需求满意度与其整体自尊水平呈显著相关。此外，张宝根等人[13]通过对初中生群体开展结构方程模型分析后发现，基本心理需求的满意度能够有效激发学生的运动意愿，推动其参与运动实践，助力运动知识与技能的掌握，同时还有利于形成积极且持久的学习行为模式。

3.4 基本心理需求、自主动机在赋予责任与课程的满意度之间存在链式中介效应

本研究结果显示，基本心理需求、自主动机在赋予学生责任与传统保健体育课程满意度之间存在链式中介效应，效应值为 0.243,占总效应的 44.57%，假设 H_5 得到支持。这些变量之间表现出显著的正向关系。有多项研究证实了自主性需求通过自主动机来影响体育课满意度及（或）愉悦感之间的关系[11]。其他一些与我们研究结果相似，如祝大鹏研究表明[9]，学生参与式体育课堂评价对提高大学生体育学习动机和学习效果

具有重要作用。像张宝根等人[13]开展的不同研究发现，如果在课堂上营造出与任务相关的适宜氛围，基本心理需求的满足感会更强，这将与更高的自我决定动机呈正相关，同时学生对体育课的厌烦情绪会减少。正如 Moreno 等人在研究中指出的，一种鼓励自主和决策的教学风格，会减少学生的消极动机以及对体育课的厌倦情绪。不同研究表明，自我决定程度较低的动机形式以及对基本心理需求满意度较低[5]，与放弃体育活动相关，这可能是由于对体育课不满所致。正如许多研究人员一段时间以来一直提议的那样[9]，有必要提出新的评价策略，让学生更多地参与其中，比如培养学生对自身行为进行反思的能力，用一个学生能从错误和成功中学习的持续过程取代期末考试，让学生参与决策等策略。基于动机层次模型的理论假设，在个体基本心理需求的满足度显著提升的前提下，自我决定程度较高的动机形式将得到多维强化，而消极动机水平则会呈现系统性下降态势。这种心理机制的良性发展将产生积极的溢出效应，不仅能够有效提升学生对体育课程的教学满意度，更可能形成行为迁移效应，显著提高其在课外场域自主开展体育活动的行为意向。

本研究的局限性，首先样本的代表性较为有限，未来研究可考虑将样本扩展至其他地区，以增强研究结果的广泛适用性。其次，本研究仅采用问卷调查的方式收集数据，由于问卷主要反映被调查者的主观态度和看法，因此在揭示行为机制方面存在局限。建议未来研究中引入方法论三角测量法，结合系统观察与定性访谈，访谈对象应涵盖学生与教师，从而获得更全面、深入的研究资料。在研究设计方面，建议后续开展纵向研究或准实验研究，通过实施具体的教学干预措施，探索变量之间的因果关系，并进一步评估不同激励策略对研究变量的影响效应。

综上所述，基于本研究所构建的模型及其分析结果，有必要在传统保健体育课程中实施有针对性的教学干预，以通过满足学生的基本心理需求来提升其自我决定动机。实现这一目标的具体策略包括：引入同伴互评机制，增强学生在教学过程中的参与感；教师在教学过程中应鼓励学生进行自我评价，并设计具有趣味性和挑战性的任务，赋予学生更多责任；同时应注重个性化教学策略的运用，设计符合学生能力水平、具有可实现挑战性的活动内容。通过上述措施，有助于提升学生对传统保健体育课程的满意度，增强其课堂自主性与积极性，并进一步促进其对体育课的积极态度和持续参与意愿。

参考文献

[1]陈善平,宋迪,谢丽君,等.中国大学生的体育锻炼动机聚类分析及体育行为特征[J].首都体育学院学报,2023,35(1): 57-67.

[2]薛锋.大学生运动动机与锻炼行为的关系:自我决定理论的视角[J].武汉体育学院学报. 2010, 44(6): 43-47.

[3]Blanchard C M, Mask L, Vallerand R J, et al. Reciprocal relationships between contextual and situational motivation in a sport setting[J].Psychology of Sport and Exercise, 2007, 8(5): 854-873.

[4]王鹏宇,刘佳宁.学校体育心理环境对青少年个人与社会责任的预测:基本心理需求的中介效应[J].沈阳体育学院学报,2020,39(4): 30-36.

[5]胡小清,唐炎,黄霞,等.基于自我决定动机理论视角下的体育学习效果研究进展[J].体育科学,2020,40(4): 67-77.

[6]郝晓亮,季浏.体育课堂决策的演进:“集权”到“赋权”：基于培养学生多种学习能力的愿景[J].西安体育学院学报. 2017,34(4): 509-512.

[7]Bechter B E, Dimmock J A, Howard J L, et al. Student Motivation in High School Physical Education: A Latent Profile Analysis Approach[J].Journal of Sport & Exercise Psychology,2018, 40(4): 206-216.

[8]万来红,陈治华,谢彬. 普通高校公共体育课学生成绩评价体系的构建与实践研究[J].武汉体育学院学报,2015, 49(12): 74-81.

[9]祝大鹏,杨华东.学生参与式体育课堂评价对大学生体育学习动机和学习效果的影响[J].浙江体育科学,2012, 34 (4): 67-71,124.

[10]葛耀君,陆遵义,卢昌亚.影响学生体育学习情境兴趣的主因素研究[J].心理科学,2012,35(2): 412-417.

[11]王琛,刘海元.欧洲 CSPAP 的学生激励措施评析与启示：基于自我决定理论[J].首都体育学院学报,2024,36(4): 435-447.

[12]Van Doren N, De Cocker K, De Clerck T, et al. The Relation between Physical Education Teachers' (De-)Motivating Style, Students' Motivation, and Students' Physical Activity: A Multilevel Approach.[J]. International journal of environmental research and public health,2021, 18(14).

[13]张宝根,唐炎,胡小清,等.基本心理需求、情境兴趣与初中生体育课堂学习投入的关系[J].体育学刊,2020, 27(2): 129-134.

[14]王景贤,于春艳.青少年体育健身运动基本心理需求满足感量表与常模制订[J].西安体育学院学报,2019,36(3): 333-341, 378.

[15]Murcia J, Coll D, Garz ó n M C. Preliminary Validation in Spanish of a Scale Designed to Measure Motivation in Physical Education Classes: The Perceived Locus of Causality (PLOC) Scale[J].Spanish Journal of Psychology,2009,12(1): 327-337.

[16]史青.大学生体育课学习满意度测查量表编制[J].体育学刊,2010,17(7): 56-60. （下转第 156 页）

的教师依据体育学科核心素养分析教材，但深度和广度上还有提升空间。45%的教师从体育学科核心素养角度分析学情，方式相对集中。60%的教师设定的学习目标与体育学科核心素养契合，但设定依据核心素养的学习目标需改进。55%的教师设计的教学流程围绕体育学科核心素养，60%的教师安排了学生自主、合作和探究学习环节。50%的教师根据体育学科核心素养选择教学策略，策略运用有一定多样性。70%的教师考虑教学环境因素，60%的教师从核心素养角度预计教学效果。70%的教师能预见到教学问题，但应对策略可能需加强。仅 45%的教师在场地器材布置回收时考虑教学任务完成，50%的教师设计的学习评价量表能全面反映学生体育学科核心素养发展水平。60%的教师确定教学重难点，65%的教师布置的体育作业与课堂教学内容紧密相关，但设计的全面性与科学性需加强。

可见，当前泉州市大多数中小学体育教师在教学设计方面存在诸多问题。一方面，教学设计的结构规范性不足，各教学要素之间的逻辑顺序较为混乱，使得教学过程缺乏清晰的脉络和合理的布局。另一方面，无论是对新课程标准精神的深度理解，还是将新理念融入教学设计的能力，亦或是在教学设计中展现创新思维和运用设计技巧方面，都存在一定程度的欠缺，均有待进一步提升和改进。

4 建议

4.1 强化教学设计专业技能培训

为全面提升中小学体育教师的教学设计能力，应定期组织专项培训。邀请教育领域的权威专家，结合丰富的教学实例，深度剖析新课程标准的核心精神，同时邀请教学成果显著的一线优秀教师分享实践经验。通过开展案例深度剖析、沉浸式模拟演练等活动，帮助教师透彻理解 “备—教—学—评一致性” 的理念，并将其有效融入教学设计，全方位助力教师提升教学设计水平。

4.2 构建多元教学研讨机制

鼓励学校或区域内的体育教师定期开展教学研讨活动，分享教学设计经验与心得，共同探讨教学过程中遇到的问题及解决方案。例如，组织集体备课活动，让教师们在交流合作中，优化教学设计结构，理清教学要素之间的逻辑关系，提升教学设计的规范性和合理性。此外，开展教学观摩活动，让教师们相互学习优秀的教学设计案例，汲取他人之长，补己之短

4.3 完善实践指导与反馈机制

学校为体育教师创造充分的教学设计实践机会，安排经验丰富、专业素养高的名师对教师进行全程跟踪指导。在教师完成教学设计后，在第一时间给出全面、细致的反馈，不仅要指出存在的问题，还要提供具体的改进策略，助力教师不断完善教学设计。同时，鼓励教师在实际教学中大胆尝试新的教学理念和设计技巧，根据课堂教学效果灵活调整教学设计，在实践中逐步提升教学设计能力。

4.4 建立健全激励机制

设立专门的教学设计奖项，对在教学设计方面表现突出的教师予以公开表彰和物质奖励，激发教师提升教学设计能力的内生动力。此外，将教学设计能力纳入教师绩效考核的重要指标体系，与职称评定、评优评先等直接挂钩，让教师切实认识到教学设计的重要性，主动投入时间和精力提升自身能力。

4.5 打造全方位资源共享平台

搭建线上线下融合的体育教学设计资源共享平台，广泛收集优秀的教学设计案例、丰富的教学设计素材、深刻的教学反思等资源，免费向教师开放。线上平台设置互动交流专区，方便教师随时交流教学设计过程中遇到的问题、分享经验与心得。线下平台则定期举办教学设计成果展示活动，展示优秀作品，为教师提供面对面学习交流的机会。

参考文献

[1]林秀春,徐瑞芳,黄倩.中小学体育教师教学设计能力调查研究[J].湖北师范大学学报(自然科学版),2018,38(1):100-107.
[2]郑华，张向东.舞动课堂 教舞育人[M].福州:福建科学技术出版社,2020:173-179.

（上接第 151 页）

[17]Hu L T, Bentler P M. Cutoff Criteria for Fit Indexes in Covariance Structure Analysis: Conventional Criteria Versus New Alternatives[J]. Structural Equation Modeling-A Multidisciplinary Journal,1999, 6(1): 1-55.
[18]Sanchez B, Byra M, Wallhead T L. Students' perceptions of the command, practice, and inclusion styles of teaching[J]. Physical Education and Sport Pedagogy,2012,17(3): 317-330.
[19]Curran T, Standage M. Psychological Needs and the Quality of Student Engagement in Physical Education: Teachers as Key Facilitators[J]. Journal of Teaching in Physical Education,2017,36(3): 262-276.
